# Statistical considerations when estimating time‐saving treatment effects in Alzheimer's disease clinical trials

**DOI:** 10.1002/alz.14035

**Published:** 2024-06-21

**Authors:** Guoqiao Wang, Gary Cutter, Neil P. Oxtoby, Guogen Shan, Whedy Wang, Brian Mangal, Yijie Liao, Jorge J. Llibre‐Guerra, Yan Li, Chengjie Xiong, Eric McDade, Paul Delmar, Randall J. Bateman, Lon Schneider

**Affiliations:** ^1^ Department of Neurology, Division of Biostatistics, School of Medicine Washington University St. Louis Missouri USA; ^2^ Division of Biostatistics Washington University School of Medicine St. Louis Missouri USA; ^3^ Department of Biostatistics University of Alabama at Birmingham Birmingham Alabama USA; ^4^ Department of Computer Science University College London London UK; ^5^ Department of Biostatistics University of Florida Gainesville Florida USA; ^6^ Tenaya Therapeutics South San Francisco California USA; ^7^ Solara Consulting Corp. North Vancouver British Columbia Canada; ^8^ Neogene Therapeutics Inc. Santa Monica California USA; ^9^ F. Hoffmann‐La Roche Ltd. Basel Switzerland; ^10^ Department of Psychiatry and The Behavioral Sciences Department of Neurology Keck School of Medicine University of Southern California Los Angeles California USA

**Keywords:** Alzheimer's disease, proportional mixed models for repeated measures, semi‐real trial data, time savings

## Abstract

**INTRODUCTION:**

Estimating treatment effects as time savings in disease progression may be more easily interpretable than assessing the absolute difference or a percentage reduction. In this study, we investigate the statistical considerations of the existing method for estimating time savings and propose alternative complementary methods.

**METHODS:**

We propose five alternative methods to estimate the time savings from different perspectives. These methods are applied to simulated clinical trial data that mimic or modify the Clinical Dementia Rating Sum of Boxes progression trajectories observed in the Clarity AD lecanemab trial.

**RESULTS:**

Our study demonstrates that the proposed methods can generate more precise estimates by considering two crucial factors: (1) the absolute difference between treatment arms, and (2) the observed progression rate in the treatment arm.

**DISCUSSION:**

Quantifying treatment effects as time savings in disease progression offers distinct advantages. To provide comprehensive estimations, it is important to use various methods.

**Highlights:**

- We explore the statistical considerations of the current method for estimating time savings.
- We proposed alternative methods that provide time savings estimations based on the observed absolute differences.
- By using various methods, a more comprehensive estimation of time savings can be achieved.

## 1 BACKGROUND

**FIGURE 1:**
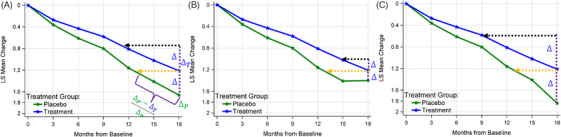
Demonstration of time‐saving treatment effects estimated by BPP and BPT methods for different scenarios (Parts A‐C). For BPP, different Δs led to the same time savings visualized by the horizontal orange line; for BPT, different Δs led to different time savings visualized by the horizontal black line. Δ represents the absolute treatment difference at 18 months, was flipped upward and subsequently projected backward onto the decline trajectory of the treatment arm in the BPT method. $\Delta_{P}$ and $\Delta_{T}$ are the mean change from baseline to Month 18 for the placebo and the treatment arm, respectively; $\frac{\Delta_{P}-\Delta_{T}}{\Delta_{P}}$ is the proportion of the difference relative to the placebo mean; $\Delta \;=\Delta_{P}\;-\Delta_{T}$. (Figure adapted from van Dyck, et al.^1^) BPP, backward projection to placebo; BPT, backward projection to treatment; LS, least squares

Clinical trials for sporadic Alzheimer's disease (AD) often use mixed models for repeated measures (MMRM) as the primary or secondary analysis model for assessing efficacy.^1^, ^2^, ^3^ This approach involves using a categorical time variable to avoid specifying the pattern of changes over time. The treatment effect is evaluated by comparing the model‐estimated change from baseline to the end‐of‐study visit (e.g., 18 months in a typical phase 3 trial) between the treatment and placebo arms. Figure 1A illustrates the estimated treatment effect with a purple vertical line (≈ 0.45 Clinical Dementia Rating Sum of Boxes [CDR‐SB^®^]), representing the absolute difference between the treatment and placebo arms.^1^ This absolute difference is often converted into a percentage slowing relative to the placebo decline (27% slowing based on the CDR‐SB in the lecanemab trial). However, ascertaining a clinically meaningful treatment effect from either the absolute difference or the percentage slowing^1^, ^3^, ^4^ is not straightforward. A more intuitive approach is to quantify the treatment effect in terms of the time savings in disease progression as demonstrated in the following.

One existing method for assessing treatment effects in terms of time savings is what we call backward projection to placebo (BPP) method.^5^, ^6^, ^7^, ^8^ Raket initially introduced this concept with applications in various contexts, as detailed here,^6^, ^7^ including: modeling raw data rather than changes from baseline, treating time as a continuous variable by using natural cubic spline trajectories for both the treatment and placebo arms, and backward projecting/matching the mean of the treatment arm to a corresponding point on the placebo arm's trajectory. Contrary to Raket's approaches, we focus on methods rooted in the MMRM framework,^8^ which use time as categorical and model the change from baseline. Essentially, this BPP method projects the estimated mean change of the treatment arm at a time point, for example, at 18 months, backward in time until it aligns with the same mean value observed in the placebo arm at an earlier time point, for example, ≈ 12.7 months in Figure 1. The time difference between these two points (18 – 12.7 = 5.3 months) represents the treatment effect measured as time savings in disease progression, visualized by the horizontal orange line in Figure 1. While as plausible as this seems, it is necessary to explain the statistical assumptions required by the BPP method and discuss how its estimated time savings can be improved. Furthermore, we will propose alternative approaches grounded in the MMRM framework for estimating time‐saving treatment effects and discuss their statistical assumptions compared to the BPP method. Three of these methods, the backward projection to treatment (BPT) method, the additional time needed to reach placebo decline relative to the placebo disease progression (ATNRP‐P) method, and ATNRP relative to the treatment disease progression (ATNRP‐T) method, focus on the single end‐of‐study time point. The fourth method uses all post‐baseline data to calculate proportional global time savings in relation to the placebo decline (PGP), using the proportional MMRM (pMMRM) model.^9^ The fifth approach similarly takes advantage of the pMMRM concept but is designed to estimate proportional global time savings compared to the treatment decline, designated as the PGT method. Comprehensive explanations of PGP and PGT methods are provided in Section 2.2.2. Furthermore, all illustrative figures will be grounded in the change from baseline, using outputs from the MMRM analysis.

**RESEARCH IN CONTEXT**

1. **Systematic review**: The authors performed an extensive literature review, using traditional sources like PubMed, alongside abstracts and presentations from scientific meetings. Through this thorough search, they successfully identified and appropriately cited relevant publications pertaining to the estimation of treatment effects as time savings.
2. **Interpretation**: In clinical trials for Alzheimer's disease, the treatment effects presented either as the absolute difference or a percentage reduction in measuring disease progression, do not lend themselves to straightforward interpretation by clinicians and patients. To enhance the interpretation, treatment effects in terms of time savings in disease progression have been used. It is necessary to explain the statistical assumptions required by the existing method and discuss how its estimated time savings can be improved. Our alternative methods can assess time savings from various perspectives by considering two crucial factors: (1) the absolute difference between treatment arms, and (2) the observed progression rate in the treatment arm. To provide comprehensive estimations, it is important to use various methods.
3. **Future directions**: To facilitate the adoption of the time‐saving concept, it is crucial to validate all approaches using real trial data from the open‐label extension period. This validation process has the potential to greatly accelerate the practical implementation of the time‐saving concept.

## 2 METHODS

### 2.1 Statistical considerations of the BPP method

We examine the statistical considerations of the BPP method from multiple angles. First, the BPP method answers the question of when the decline in the treatment arm, at 18 months, becomes equivalent to that estimated in the placebo arm. However, it does not directly address the extent to which disease progression has been delayed (i.e., time savings), because it is not compared to the placebo arm over the 18‐month treatment period and does not account for the absolute treatment effects at 18 months. As an example, consider Figure 1A–C, in which all demonstrate identical time‐saving effects of 5.3 months. However, it is important to note that these figures display significant variations in placebo decline between months 15 and 18, resulting in different absolute treatment effects (Δ at 18 months). This notable inconsistency between the various absolute treatment effects and the consistent time‐saving treatment effect complicates the interpretation of the time‐saving effect and offers varying interpretations of the study results based on when the comparisons are made. Second, the assumptions underlying the Figure 1 5.3‐month time savings are critical. For one, it assumes that, in the absence of treatment, the treatment arm would have declined at the same rate as the placebo arm. This is unlikely to be true due to the underlying factors such as differences in the in‐study follow‐up durations (18 vs. 12.7 months) and the heterogeneity of response between the two arms. Furthermore, this assumption is not testable. Participants receiving the active drug typically will continue treatment in the open‐label extension (OLE) period when the treatment is effective during the double‐blind phase.^10^ Consequently, we cannot assess whether the treated participants in the absence of treatment will experience the same rate of decline as those on placebo. Third, the backward comparison between the treatment arm at 18 months and the placebo arm at ≈ 12.7 months disrupts the randomization equilibrium due to the subsequent dropouts over time and the unequal dropouts between treatment arms. Thus, this comparison across arms at different time points is only valid under missing completely at random or when there are no missing data. The missing at random assumption will not hold because the observed information between the linked time points are not equal and the time variable is not equal. To illustrate, Figure S1 in supporting information presents the dropout rates over time across treatment arms observed in the lecanemab^1^ and the donanemab^3^ trials both with a 1:1 randomization ratio. The circles indicate the linked time points based on the BPP method. The difference in the dropout rates between the matched time points are approximately 5.6% (16.9% at 18 months vs. ≈ 11.3% at ≈ 12.7 months) for lecanemab and 9.7% (22.3% at 76 weeks vs. ≈ 12.6% at 46 weeks) for donanemab. It seems reasonable to argue that the differential dropout rates lead to non‐comparable cohorts between the participants who reached the 18‐month/76‐week mark in the treatment arms and the earlier time point in the placebo arms. Specifically, we might see that participants staying on study until 18 months are either in better health or more tolerant to the treatment than those at 12.7 months/46 weeks in the placebo arm. This then potentially results in a slower rate of decline compared to the placebo participants. Finally, the BPP method backward projects the mean change at the single end‐of‐study time point of the treatment arm to the placebo decline trajectory. It is not a global approach, as it does not incorporate a comparison of the entire post‐baseline disease progression trajectories between the treatment and placebo arms. To more accurately assess the extent of disease progression delay after an 18‐month treatment, several factors should be taken into consideration: (1) the difference in disease progression at the end of a study (e.g., 18 months for the lecanemab trial) between the treatment and placebo arms, (2) the rate of disease progression among participants receiving treatment, (3) the testability of the assumption on the disease progression rate used in the statistical model, (4) the entire post‐baseline disease progression trajectories. The BPP method does not sufficiently address these essential factors. We offer alternative methodologies that can mitigate the constraints of the BPP method by effectively addressing these factors.

### 2.2 Alternative methods to estimate time savings in disease progression

#### 2.2.1 Single end‐of‐study time point approach

To facilitate comparison to the BPP method, we first present three approaches that focus on the mean change from baseline at the end‐of‐study timepoint.

##### 2.2.1.1 Backward projection to treatment (BPT) Method

To account for factors (1) to (2) described in the Section 2.1, we first propose the BPT method as a solution (Figure 1). This approach entails projecting the absolute treatment difference at 18 months (Δ in Figure 1) backward in time, aligning it with the decline trajectory of the treatment arm until the treatment arm decline matches the observed difference, as illustrated by the horizontal black line. This method leverages the information (Δ) obtained at the same visit (e.g., 18 months), thereby maintaining a comparable cohort comparison by not matching different time points from different arms. Further, as participants receiving treatment usually continue into the OLE period when the treatment is effective during the double‐blind phase, it becomes possible to test the assumption on the disease progression rate used by the BPT method, which is to compare disease progression rates before and after the 18‐month time point using additional data from the OLE period.

The interpretation of the estimated time savings using this method is as follows: considering the observed disease progression rate under treatment and the observed reduction in disease progression (i.e., the absolute treatment effect), while participants are on treatment, how much longer has the treatment delayed the disease progression compared to the decline seen in the placebo arm (in other words, how much longer does it take to reach the same level of decline as observed in the placebo arm while under treatment)? This method offers a more precise, pragmatic, and direct estimation of time savings in disease progression by integrating both the observed treatment trajectory and the difference observed at the study's particular and common time point. It assumes that the time savings should be estimated when the participants are under the same treatment condition. Furthermore, the BPT method estimates the time savings based on the most recent disease progression rate observed throughout the treatment period. This approach can effectively capture both disease‐modifying and symptomatic treatment effects, making it a versatile tool for evaluating treatment outcomes. Figure 1 illustrates the estimated time savings for identical disease progression patterns using both BPP and BPT methods. Unlike the estimates provided by BPP, the time savings derived from the BPT method reflect the variations in the estimated absolute treatment effects (∆ at 18 months).

##### 2.2.1.2 Proportional/percentage treatment effect and time savings

The more traditional efficacy inference replies on the absolute difference in the primary endpoint between the treatment and placebo arms, measured on the *y* axis, often translated into a proportional or percentage reduction relative to placebo decline (e.g., $\frac{\Delta_{P}-\Delta_{T}}{\Delta_{P}}$ in Figure 1A).^1^, ^3^ In contrast, time savings quantify a difference along the time variable, measured on the *x* axis, which can be similarly expressed as a proportional or percentage relative to the total trial duration. Indeed, the immediate result from Raket's proportional time models is a proportion estimated based on the backward projection concept rather than a direct measure of time savings.^7^ We will show that the absolute difference and time savings ultimately can be approximated by the same proportional concept.

As visualized by the horizontal orange line in Figure 1A, the foundational concept of the BPP method is that, within the trial duration, if the placebo shows a decline of $\Delta_{P}$ and the treatment shows a decline of $\Delta_{T}$, then the duration required for the placebo to decline from $\Delta_{T}$ to $\Delta_{P}$ represents the time savings throughout the trial. Therefore, the proportion of the time savings relative to the total trial duration can be approximated as $\theta_{T\_PBO}=\frac{\Delta_{P}-\Delta_{T}}{\Delta_{P}}\;$, where $\theta_{T\_PBO}$ means BPP measures the proportion of time savings relative to the placebo decline trajectory. This proportion mirrors the proportional reduction depicted on the *y* axis at the end‐of‐study visit. On the other hand, with the BPT method, this proportion is similarly approximated as $\theta_{T\_TX}=\frac{\Delta_{P}-\Delta_{T}}{\Delta_{T}}\;$, where $\theta_{T\_TX}$ means the proportion of time savings is measured relative to the treatment decline trajectory. This, too, corresponds to a proportional increase of decline in the placebo arm on the *y* axis relative to the treatment decline at the end‐of‐study visit.

**FIGURE 2:**
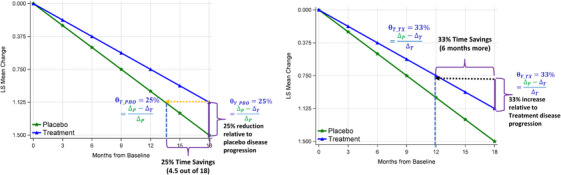
Demonstration of the equivalent proportional treatment effect between the absolute difference on *y* axis and the time savings on *x* axis. Left: BPP method; Right: BPT method. BPP, backward projection to placebo; BPT, backward projection to treatment; LS, least squares

The fact that the proportional treatment effect, whether measured on the *y* axis (as absolute difference) or on the *x* axis (as time savings), being approximated by the same formula, becomes clearer with linear disease progression trajectories, as shown in Figure 2. With linear progression, these proportions are equivalent. Consequently, we suggest calculating time savings using the proportional treatment effect, as determined from the absolute difference on the *y* axis at the end‐of‐study visit or across all or multiple post‐baseline visits, in the subsequent section.

**FIGURE 3:**
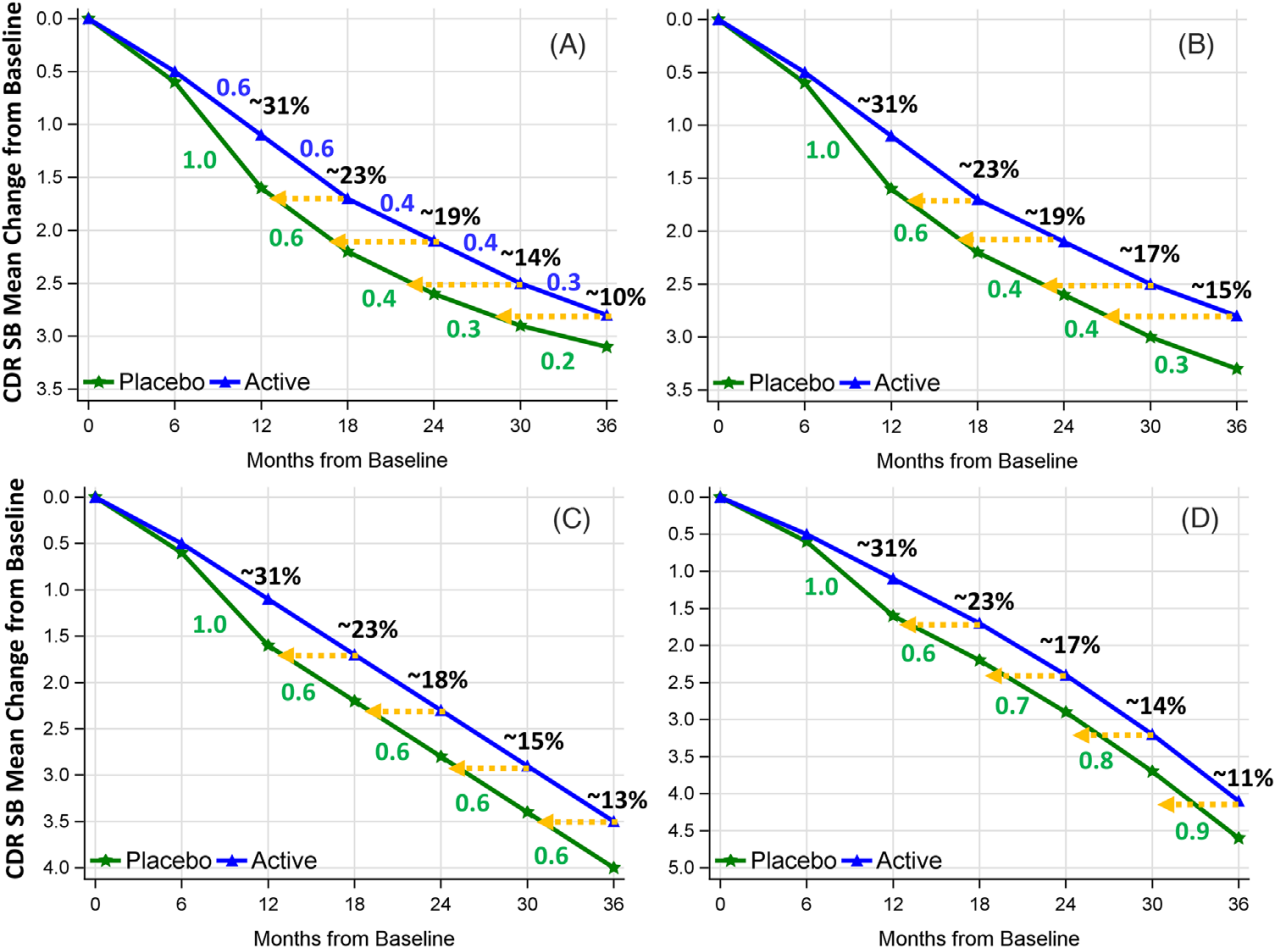
Illustration of time savings across various scenarios (Parts A‐D). The percentages, like ≈ 31% in black, represent the proportional reduction in disease progression at each visit compared to the decline in the placebo arm starting from Month 12. The numbers in green color, such as 1.0 and 0.6, indicate the decline observed in the placebo arm over each 6‐month follow‐up interval. In panels 3B, 3C, and 3D, both the placebo and treatment arms exhibit identical declines over each follow‐up interval starting from Month 12. The numbers in blue like 0.6 in Figure 3A, denote the decline in the treatment arm during each 6‐month follow‐up interval. (Figure 3A was recreated based on Raket's MMRM Figure,^6^ and Figure 3B was adapted from Petersen et al.^5^ The values such as the mean change from baseline to each visit were approximately extracted from their respective figures, thus may be slightly different from those used in their figures). CDR‐SB, Clinical Dementia Rating Sum of Boxes; MMRM, mixed models for repeated measures

For methods like BPP, which do not use the absolute difference ($\Delta_{P}-\Delta_{T}$), the proportion of time savings may not align with the proportional reduction shown on the *y* axis at the end‐of‐study visit under certain conditions, as demonstrated in Figure 3 and Table S1 in supporting information. In this figure, based on the BPP method, time savings of 8.0 months (22% of the total 36‐month duration) are achieved at month 36 for Scenario 3A, 9.0 months (25%) for 3B, 5.0 months (14%) for 3C, and 3.3 months (10%) for 3D. With the BPP method, the trajectory of placebo decline has a substantial impact on the estimated time savings duration. For instance, in Scenario 3A, the placebo's progression every 6 months improves from 1.0 units to 0.2 units, becoming less than the active treatment's progression from Month 24 to 36 (0.4 vs. 0.3 from Month 24 to 30 and 0.3 vs. 0.2 from Month 30 to 36). In Scenario 3B, the placebo's progression gradually reduces from 1.0 units to 0.3 units but matches the active treatment's progression rate from Month 12 to 36. Scenarios 3C and 3D see both arms declining at the same rate from Month 12 to 36, without a decrease in progression over each 6‐month interval. The BPP method's reliance on placebo decline trajectory means that as the placebo progression rate increases from 3B to 3D, the time savings proportions relative to the total duration decrease from 25% to 10%. The faster the decline, the shorter the time savings. When the placebo decline rate does not decrease (Scenarios 3C and 3D), the proportion of time savings closely matches the proportion of reduction estimated by $\frac{\Delta_{P}-\Delta_{T}}{\Delta_{P}}$, with 14% versus 13% for 3C and 10% versus 11% for 3D. It's important to note that even though the placebo decline may stabilize in CDR‐SB over each follow‐up interval in both the placebo and treatment arms, as seen in the Clarity AD lecanemab trial^1^ and the TRAILBLAZER‐ALZ 2 donanemab trial^3^ (Table S2 in supporting information), there are no reports of the placebo decline being equal to (in 3B) or smaller than (in 3A) the treatment decline in CDR‐SB over several follow‐up intervals for disease‐modifying treatments. Indeed, in hypothetical Scenarios 3A and 3B, assuming other variables like dropout rates and mechanisms of missing data are consistent across both arms, one could argue that the treatments exhibit merely symptomatic effects or are even less beneficial than placebo, making the time savings less relevant regardless of magnitude. On the other hand, these scenarios could result from differential dropout rates between the placebo and treatment groups due to data not missing at random. For instance, a higher proportion of participants in the placebo arm might withdraw due to more pronounced disease progression from not receiving treatment. Consequently, those who continue in the placebo arm might be healthier and thus exhibit a less significant decline compared to those remaining in the treatment arm toward the trial's latter stages. However, it is commonly observed that the dropout rate in the placebo arm is lower than that in the treatment arm in most AD clinical trials, regardless of the treatment effect.^1^, ^3^, ^11^, ^12^ This trend suggests that Scenarios 3A and 3B are unlikely when the treatment under investigation is disease modifying.

##### 2.2.1.3 Additional time needed to reach the placebo decline (ATNRP) method

Major benefits of estimating time savings through the proportion derived from $\Delta_{P}$, $\Delta_{T}$, and the difference ($\Delta_{P}-\Delta_{T}$) rather than using backward projection include: (1) maintaining the randomization equilibrium, as both estimates are derived during the same visit; (2) accounting for the magnitude of the absolute difference. It is also intuitive to interpret: Given the observed reduction in disease progression between the treatment and placebo arms over the trial duration of $T_{total}$, what is the additional time needed for participants in the treatment arm to reach the placebo decline observed by the trial's end if they were to stop the treatment ($T_{PBO}=\theta_{T\_PBO}\;\times T_{total}$) or continue with the treatment ($T_{TX}=\theta_{T\_TX}\;\times T_{total}$)? $T_{PBO}$ is estimated assuming that, in the absence of treatment, the initial treatment arm will decline at a rate similar to the placebo arm as observed during the trial (termed as ATNRP‐P), an assumption also used in the BPP method, where $\theta_{T\_PBO}=\frac{\Delta_{P}-\Delta_{T}}{\Delta_{P}}\;$. ATNRP‐P uses a proportional time‐saving concept grounded in the proportional reduction in the change from baseline observed at the end‐of‐study visit relative to the placebo decline. The underlying rationale is as follows: If, throughout the trial, the treatment arm demonstrates a proportional reduction in disease progression relative to the placebo arm, quantified as $\theta_{T\_PBO}$, then the additional time required to achieve the same level of decline as the placebo should be proportional to the overall duration of the trial. This can be calculated using the formula ($T_{PBO}=\theta_{T\_PBO}\;\times T_{total}$).

Conversely, the calculation of $T_{TX}$ assumes that with the ongoing treatment, the initial treatment arm will continue to decline in a rate similar to that observed within the treatment arm during the trial (termed as ATNRP‐T), an assumption also integral to the BPT method, where $\theta_{T\_TX}=\frac{\Delta_{P}-\Delta_{T}}{\Delta_{T}}\;$. ATNRP‐T also uses a proportional time‐saving concept though relative to the treatment decline. Therefore, like the BPT method, this assumption can be validated by comparing the disease progression rates before and after the end‐of‐study time point (e.g., 18‐month time point in many AD trials) using OLE data.

Because both $\theta_{T\_PBO}$ and $\theta_{T\_TX}$ derived from the same dataset, they are inherently connected by the formula:

$$\;\theta_{T\_TX}=\frac{\theta_{T\_PBO}}{1-\theta_{T\_PBO}}\;=\left(\frac{1}{1-\theta_{T\_PBO}}-1\right)\;.$$

For example, lecanemab led to a 27% ($\theta_{T\_PBO}$) reduction in disease progression at Month 18 in the Clarity AD trial and can be used as an approximation for future treatment effects beyond 18 months. The extra time in months needed to reach the level of placebo decline at 18 months can then be estimated as:

$$\;T_{PBO}=\theta_{T\_PBO}\;\times 18\;=\;27\%\times 18\approx 4.9.$$

Or as:

$$\;T_{TX}=\theta_{T\_TX}\;\times 18\;=\left(\frac{1}{1-\theta_{T\_PBO}}-1\right)\;\times 18\approx 6.7.$$

The discrepancy in estimated additional time needed to reach the placebo decline arises from the distinct assumptions underlying each method.

#### 2.2.2 Global approaches using all post‐baseline visits

**FIGURE 4:**
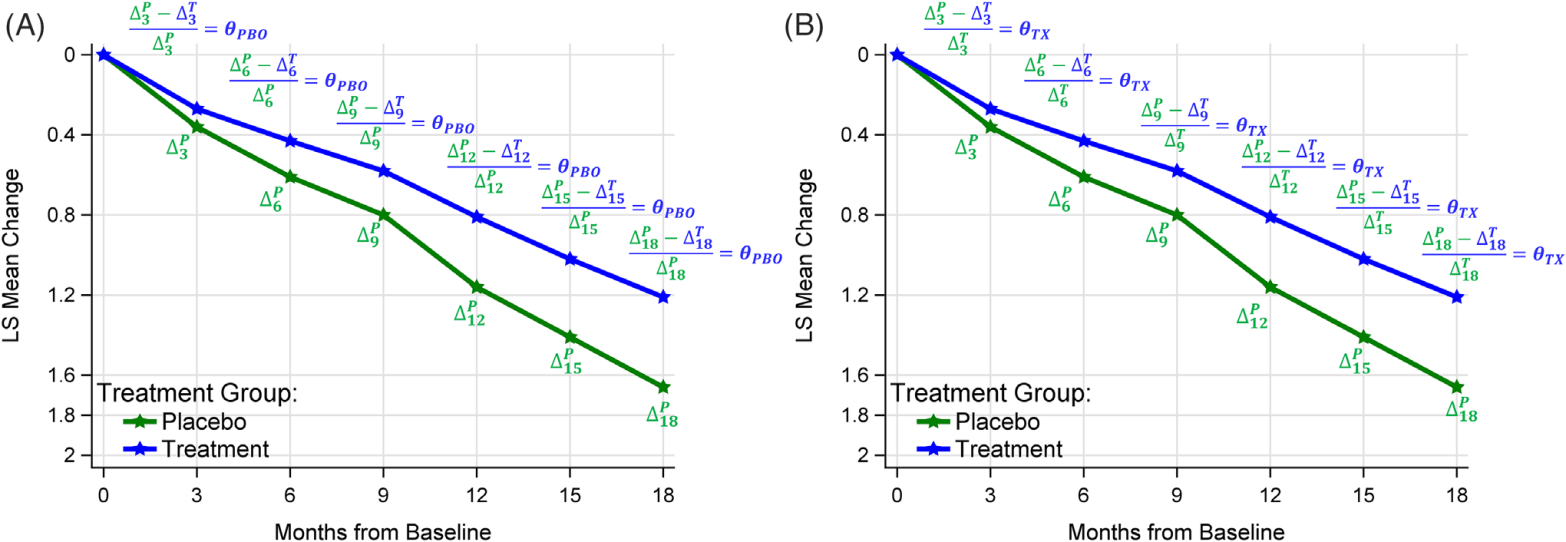
Demonstration of the global approach relative to the placebo decline (Part A) or the treatment decline (Part B) across all post‐baseline visits. Part A: proportional reduction relative to the placebo decline; Part B: proportional increase relative to the treatment decline. $\Delta_{j}^{P}$ and $\Delta_{j}^{T}$ represents the mean change from baseline to Month j of the placebo and treatment arms, respectively. $\theta_{PBO}$ and $\theta_{TX}$ represent the proportional treatment effects over the entire trial duration with the place decline as the reference and the treatment decline as the reference, respectively. (Figure adapted from van Dyck, et al.^1^) LS, least squares

The connection between the time savings and the mean change from baseline $\Delta_{P}$, $\Delta_{T}$, and their difference ($\Delta_{P}-\Delta_{T}$) through the proportional metric provides an intuitive way to develop global approaches to estimate time savings across the entire post‐baseline disease progression trajectories. This strategy aligns with the concept of pMMRM, as depicted in Figure 4. Instead of deriving this metric solely from the end‐of‐study time point, the pMMRM‐based technique leverages all post‐baseline visits to compute an average metric, whether compared to the placebo's decline (Figure 4A) or in relation to the treatment's decline (Figure 4B). We term these methodologies PGP and PGT, respectively. Similar to the ATNRP methods, the proportional time‐saving concept can be applied to compute time savings using the proportion estimated across all post‐baseline visits. This is formulated as:

$$\;T_{PBO\_Overall}=\theta_{PBO}\;\times 18,$$

when referencing the decline in the placebo group;

Or as:

$$\;T_{TX\_Overall}=\theta_{TX}\;\times 18,$$

when the treatment decline serves as the reference.

These global approaches stand in contrast to Raket's method in: (1) avoiding backward projection (horizontal comparison) by estimating the proportion using the observed mean change from baseline at the same visit (vertical comparison); (2) using time as a categorical variable, rather than treating it as a continuous variable; and (3) assuming unstructured disease progression trajectories rather than natural cubic spline–based trajectories. It is noted that the proportional treatment effect does not necessarily need to be estimated across all post‐baseline visits. Instead, it can be estimated using only the last few visits (Figure S2 in supporting information) after any meaningful practice effects or short‐term substantial symptomatic effects has subsided.^3^, ^9^, ^13^

### 2.3 Estimation method

All these methods for estimating time savings can be implemented using an intuitive two‐step approach. For methods like BPP, BPT, ATNRP‐P, and ATNRP‐T, the initial step involves running the conventional MMRM model to derive the estimated least squares mean changes from baseline and their corresponding variance–covariance matrix. The subsequent step uses the delta method to calculate the time‐saving parameter and its variance, in addition to the 95% confidence interval.^3^, ^14^ For global approaches like PGP and PGT, the proportional (%) treatment effect is initially determined using the pMMRM approach, which is then translated into time savings by multiplying it by the total trial duration. All methodologies were implemented using SAS software.

## 3 RESULTS

### 3.1 Application to semi‐real trial data mimicking the clarity AD lecanemab trial

#### 3.1.1 Impact of varied placebo disease progression trajectories on time savings

Because BPP does not account for absolute treatment effects (or the placebo decline rate) beyond the matched time point, to evaluate the influence of varied placebo disease progression trajectories on time savings for each method, three semi‐real clinical trials were simulated. These trials, illustrated in Figure 1, were simulated mimicking the disease progression trajectories observed in the Clarity AD lecanemab trial as closely as possible. This strategy of evaluating the time‐saving treatment effect over time, through varying the placebo disease progression trajectories, was adopted previously by Petersen et al.^5^ in reference to Rachet's original figure.^6^

The semi‐real trial data were simulated, and upon analysis using the MMRM model, the published disease progression trajectories based on real lecanemab trial data could be reproduced (Figure 1A). Hence, they are denoted as semi‐real trial data. The simulated data allow us to obtain the estimation variability for each method and to compare to each other. It is important to note that while these simulations offer useful datasets for estimating the variability, the results derived from them should not be directly compared to real trial data. The simulated data may not fully capture the complexities and nuances of actual trial data and potentially lead to estimates that differ slightly from those derived from actual trial data, making direct comparisons inappropriate.

Briefly, the simulation is set up in the following way:

1. The LS mean changes from baseline in CDR‐SB were primarily derived from the figure of the lecanemab trial 1, except at 18 months where they were explicitly provided (Table S3 in supporting information).
2. The placebo LS mean at 18 months was subsequently modified (either decelerated [Figure 1B and Table S3] or accelerated [Figure 1C and Table S3]) to assess model performance. These hypothetical scenarios provide insight into the robustness of model estimations.
3. The covariance matrix (6 by 6) for changes from baseline was extrapolated from the estimated variance at the 18‐month mark and is assumed to be the same for both the placebo and treatment arms. The covariance component was computed using the variances at each time point and a first‐order heterogeneous autoregressive correlation matrix of 0.8.
4. The sample size (Table S3) and dropout rates (Figure S1, left panel) for each post‐baseline visit in each arm were aligned with those observed in the lecanemab trial 1.

**FIGURE 5:**
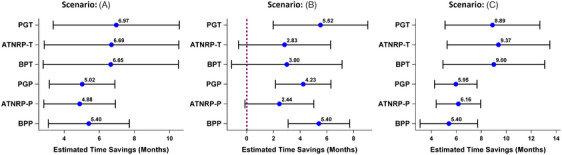
Estimated time savings and 95% confidence intervals by different methods for various scenarios (Parts A‐C). ATNRP‐P, additional time needed to reach the placebo decline relative to the disease progression rate observed in the placebo arm; ATNRP‐T, additional time needed to reach the placebo decline relative to the disease progression rate observed in the treatment arm; BPP, backward projection to the placebo decline; BPT, backward projection to the treatment decline; PGP, proportional global time savings relative to placebo; PGT, proportional global time savings relative to treatment

The disease progression trajectories of the three simulated clinical trials and the demonstration of how to estimate the time savings have been depicted in Figure 1. The details of the estimated time savings for each method are presented in Figure 5 and Table S4 in supporting information.

In all scenarios, BPP consistently yielded the same estimate of time savings due to its lack of incorporation of treatment differences at 18 months (Figure 5). Conversely, all the other methods produced varying estimates of time savings across different scenarios, reflecting the treatment differences at 18 months and the necessity of using more post‐baseline data. When the treatment effect, as indicated by the absolute difference between arms, continues to increase (illustrated in Figure 1A and Figure 1C), the estimated time savings demonstrate greater consistency within each method cluster (based on placebo progression: BPP, ATNRP‐P, PGP vs. based on treatment progression: BPT, ATNRP‐T, PGT; Figure 5). In these scenarios, the placebo arm's faster decline compared to the treatment arm leads to shorter time savings estimates by the methods (BPP, ATNRP‐P, PGP) that base calculations on placebo disease progression, in contrast to those (BPT, ATNRP‐T, PGT) that are derived from the treatment disease progression. Conversely, when the treatment effect diminishes over time, as seen in Figure 1B, there is more variability in the estimated time savings (Scenario: Figure 1B in Figure 5). This variability stems from the distinct assumptions and the type of information each method uses. For example, the BPP method does not incorporate the treatment difference at 18 months, leading it to calculate time savings using a smaller number of mean changes compared to the BPT method. Consequently, this results in estimated time savings that exhibit less variability compared to those estimated by the BPT method.

The estimated mean changes from baseline for MMRM and pMMRM methods are presented in Table S5 in supporting information, and the estimated disease progression trajectories from each method were overlayed and demonstrated in Figure S3 in supporting information. Despite the use of a proportional treatment effect, pMMRM demonstrates strong consistency with MMRM's trajectories. Notable deviation occurred when the treatment effect faded at the 18‐month visit (Figure S3 panel B), with the largest discrepancy of 0.09 in the progression trajectories of the treatment arms.

#### 3.1.2 Comparison of estimated time savings across different treatment effect scenarios

**FIGURE 6:**
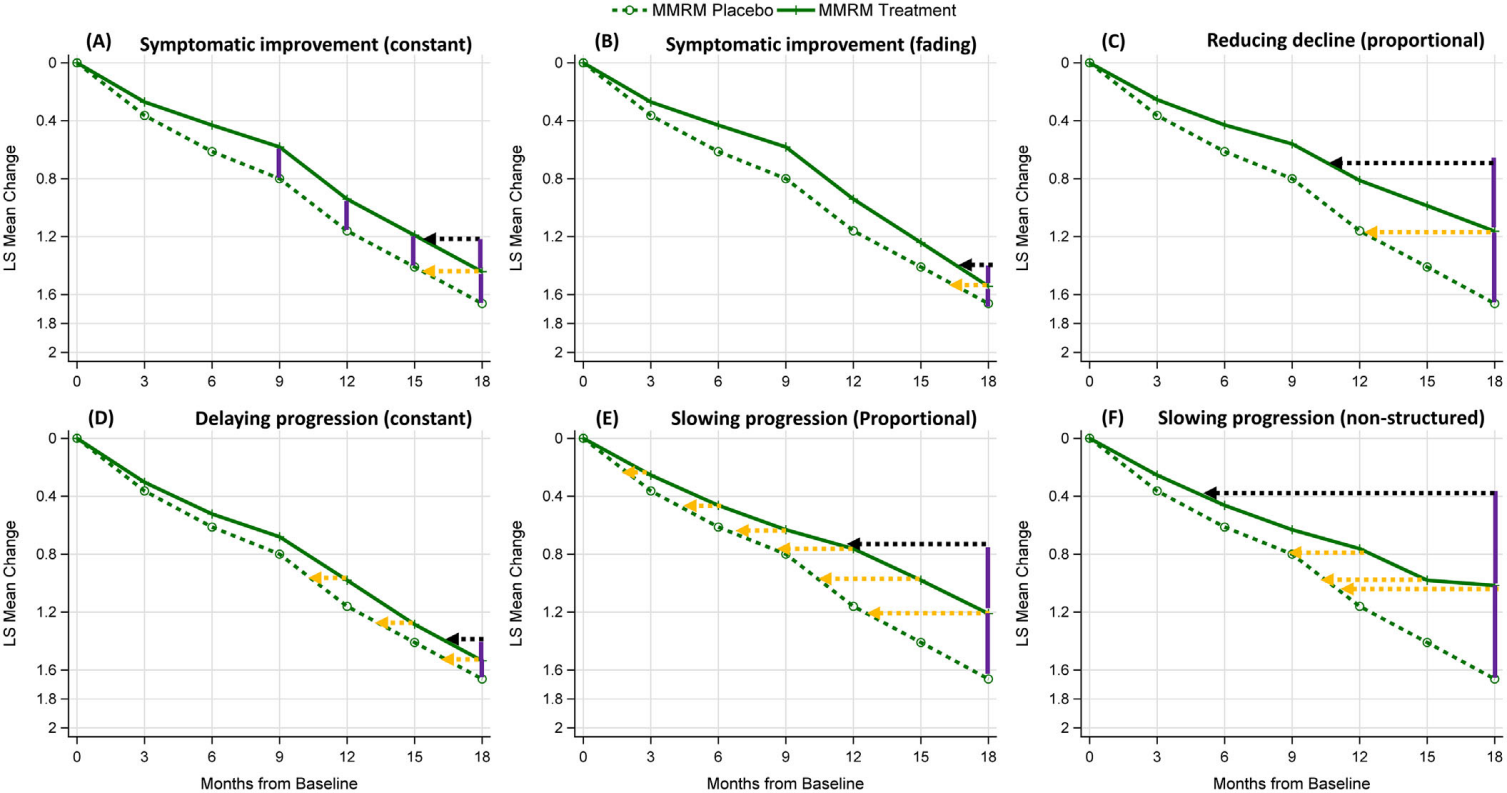
Illustration of various treatment effects by modifying the Clarity AD lecanemab disease progression trajectories (Parts A‐F). A, The symptomatic treatment effect incrementally develops from baseline and culminates at Month 9, quantified as a difference of 0.219. Beyond this point, it maintains a constant vertical separation between the curves, extending from Month 9 to Month 18, as illustrated by the vertical purple lines of equal length. B, The symptomatic treatment effect builds from baseline to Month 12, quantified as a difference of 0.219, then decreases slightly to 0.169 at Month 15, and to 0.119 at Month 18. C, The treatment effect is evidenced by a proportional reduction (30%, as depicted in this specific figure) in the disease progression of the placebo arm, resulting in a consistent ratio (70%) of the treatment arm relative to the placebo arm in terms of mean change from baseline across all post‐baseline visits. D, The treatment effect of delaying progression in time develops from baseline to Month 12, and then remain as a constant delay in time, quantified based on the BPP method as 1.5 months at Months 12, 15, and 18, as illustrated by the horizontal orange lines of equal length. E, The treatment effect of slowing progression is evidenced by a proportional reduction in time to decline (30%, as depicted in this specific figure), as illustrated by the horizontal orange lines of increasing length (0.9 months based on the BPP method at Month 3, 1.8 at Month 6, 2.7 at Month 9, 3.6 at Month 12, 4.5 at Month 15, 5.4 at Month 18). F, The treatment effect of slowing progression follows an unstructured pattern, as illustrated by the horizontal orange lines of different lengths. BPP, backward projection to the placebo; LS, least squares; MMRM, mixed models for repeated measures

To enhance the comparison of model performance in estimating time savings, various treatment effect scenarios proposed by Raket were generated by altering the disease progression trajectories observed in the Clarity AD lecanemab trial (Figure 6; Table S6 in supporting information).^1^ Contrary to Raket's method,^7^ these trajectories are derived from modifications of real‐trial MMRM outputs, treating time as a categorical variable instead of using hypothetical natural cubic spline trajectories with time as a continuous variable. As a result, these curves are less smooth than the ones Raket proposed.

Estimated time savings for each scenario, as calculated by various methods, are detailed in Figure S4 and Table S7 in supporting information. For symptomatic treatment effects (Scenarios A, B, D), all methods produced very similar estimated time savings. In cases of disease‐modifying treatment effects, characterized by increasing separation between the arms (Scenarios C, E, F), methods that calculate based on placebo disease progression (BPP, ATNRP‐P, PGP) generated shorter estimated time savings with narrower confidence intervals. Conversely, methods based on treatment disease progression (BPT, ATNRP‐T, PGT) resulted in longer estimates accompanied by broader confidence intervals. This outcome is reasonable because all methods derive time savings from the same dataset but apply distinct assumptions regarding disease progression beyond 18 months of treatment.

**FIGURE 7:**
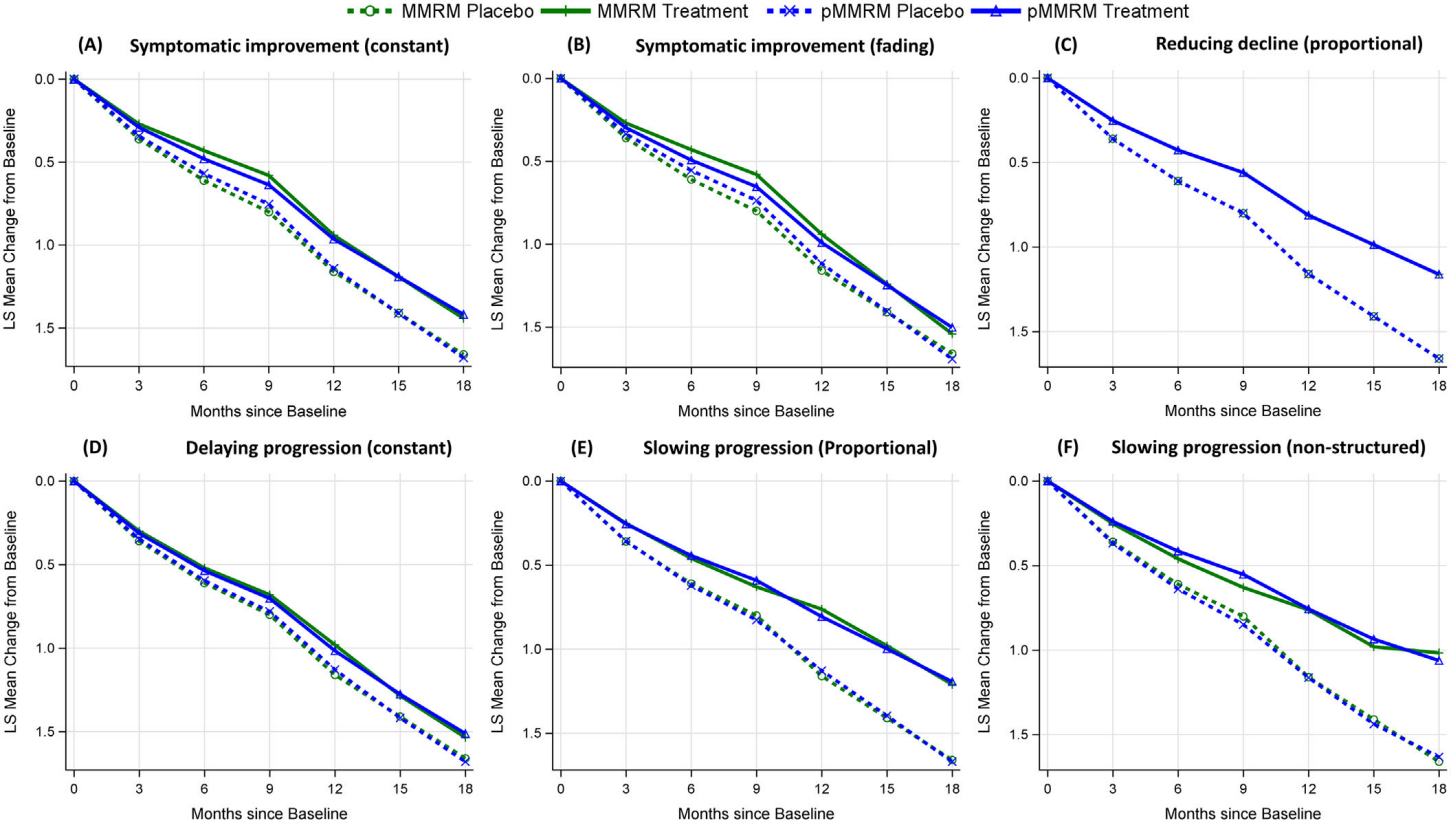
Comparison of the estimated disease progression trajectories between MMRM and pMMRM methods for various hypothetical scenarios (Parts A‐F). Both MMRM and pMMRM were applied to the same simulated data. LS, least squares; MMRM, mixed model for repeated measures; pMMRM, proportional MMRM.

Figure 7 illustrates a comparison of the estimated disease progression trajectories between the MMRM and pMMRM methods (depicted in Figure 4A) by superimposing one model's output over the other's. Despite the proportionality assumption, the pMMRM demonstrated resilience against assumption violations, particularly at the last visit, where its results closely aligned with those from the MMRM. Table S8 in supporting information presents the estimated mean changes from baseline from both models. The most significant discrepancies are generally seen at the middle visit(s), with the greatest deviation of 0.08 recorded at Month 9 in the “Slowing progression (non‐structured)” scenario. The tight alignment between disease progression trajectories as generated by the MMRM and pMMRM methods suggests that the proportional treatment effect calculated by pMMRM can serve as a reliable measure of the treatment's impact over the entire duration of the trial.

### 3.2 Summary and conceptual comparison of various methods

Summary and conceptual comparisons of the methods are outlined in Table 1. The key distinctions between these methods include: (1) the choice of disease progression trajectory (placebo vs. treatment) as the reference for estimating time savings; (2) the extent of post‐baseline data use (backward project the end‐of‐study visit or only use the difference at the end‐of‐study visit vs. all post‐baseline visits).

**TABLE 1:** Summary of assumptions and limitations for each method.

Time points	Method	Assumptions/limitations
End‐of‐study visit	BPP	**1**. Disrupts the randomization equilibrium due to increased dropout rates over time and different dropout rates across treatment arms (i.e., comparison between treatment and placebo is conducted at different time points). **2**. Only valid under missing completely at random due to the match of different time points. **3**. Inconsistency between the estimated time savings and the absolute differences due to the lack of use of the observed difference at the end of study (i.e., time savings do not reflect the absolute difference). **4**. Use of the placebo disease progression rate requires that treated participants after a longer follow‐up (e.g., after 18 months) will decline at the same rate as the placebo participants after a shorter follow‐up (e.g., after 12.7 months). This assumption is not testable in a real trial since all participants will receive the active drug in the OLE period.
	BPT	**(i)** No disruption of randomization equilibrium since comparison between treatment and placebo is conducted at the same time points **(ii)** Valid under missing at random **(iii)** Time savings reflect the absolute differences **(iv)** Use of the treatment disease progression rate before the end of study (i.e., 18 months). The disease progression rate before and after the end of study can be compared using OLE data
	ATNRP‐P	**(i)–(iii) the same as BPT** **(iv)** in the absence of treatment, the initial treatment arm after the end of study (e.g., 18 months) will decline at a rate similar to that observed within the placebo arm during the trial before the end of study (e.g., 18 months). Use a proportional time saving concept based on the proportion estimated at the end‐of‐study visit
	ATNRP‐T	**(i)–(iii) the same as BPT** **(iv)** with the ongoing treatment, the initial treatment arm after the end of study (e.g., 18 months) will decline at a rate similar to that observed within the treatment arm during the trial before the end of study (e.g., 18 months). Use a proportional time saving concept based on the proportion estimated at the end‐of‐study visit
All post‐baseline visits	PGP	**(i)–(iii) the same as BPT** **(iv)** in the absence of treatment, the initial treatment arm after the end of study (e.g., 18 months) will decline at a rate similar to the **overall average** rate observed within the placebo arm during the trial before the end of study (e.g., 18 months). Use a proportional time saving concept based on the proportion estimated across all post‐baseline visits
	PGT	**(i)–(iii) the same as BPT** **(iv)** with the ongoing treatment, the initial treatment arm after the end of study (e.g., 18 months) will decline at a rate similar to the **overall average** rate within the treatment arm during the trial before the end of study (e.g., 18 months). Use a proportional time saving concept based on the proportion estimated across all post‐baseline visits

Abbreviations: ATNRP‐P, additional time needed to reach the placebo decline relative to the disease progression rate observed in the placebo arm; ATNRP‐T, additional time needed to reach the placebo decline relative to the disease progression rate observed in the treatment arm; BPP, backward projection to the placebo decline; BPT, backward projection to the treatment decline; PGP, proportional global time savings relative to placebo; PGT, proportional global time savings relative to treatment.

## 4 DISCUSSION

Quantifying treatment effects as time savings in disease progression could make the treatment effect more easily interpretable, appealing to both clinicians and patients when evaluating clinical meaningfulness. Raket suggested estimating the time‐saving effect by: (1) modeling disease progression trajectories using natural cubic spline with time as a continuous variable; (2) backward projecting/matching the treatment arm's mean decline until it aligns with the placebo arm's decline (horizontal comparison, termed BPP for simplicity); and (3) modeling raw values.^7^ Raket's proposal includes both single time point and global approaches, including proportional time savings. Building on the concept of backward projection to the placebo, Dickson et al. proposed estimating the time effect using outputs from the established MMRM analysis, treating time as a categorical variable.^8^ We expanded on their work by introducing alternative methods that use different techniques for estimating time savings and rely on less stringent statistical assumptions (Section 3.2, Table 1). Our methods differ from Raket's in several aspects: (1) time is treated as categorical, making the disease progression trajectory unstructured and not confined to a natural cubic spline shape; (2) our global approaches calculate the overall proportional treatment effect by vertically comparing mean changes from baseline between the treatment and placebo arms at each visit,^9^ as opposed to Raket's horizontal, matching comparisons; (3) we focus on modeling the change from baseline rather than raw values; (4) all methods use the differences in mean change between treatment and placebo arms either at the end‐of‐study visit or across all post‐baseline visits. We demonstrated that whether estimating treatment effects on the *y* axis (difference in mean changes) or time savings on the *x* axis, when translated into a proportional treatment effect (i.e., % of time savings relative to the total duration vs. % of reduction due to the active treatment compared to the placebo progression), these effects tend to converge to the equivalent or similar proportion (Section 2.2.1). Given that all treatment effects are derived from the same dataset, this inherent link among different manifestations of treatment effects is both expected and reassuring. Consequently, both our approaches (ATNRP‐P, ATNRP‐T, PGP, PGT) and some of Raket's first provide a proportional treatment effect estimate before converting it to time savings (i.e., the direct model output is a proportion).

Among the six methods evaluated, two (PGP and PGT) are global approaches that use all post‐baseline data, while the other four are single time point methods focusing either on the mean change at the end‐of‐study visit (BPP and BPT) or the proportional treatment effect observed at that visit (ATNRP‐P and ATNRP‐T). From an estimand framework perspective, global and single time point approaches address different clinical questions. Assuming an 18‐month trial duration, the former evaluates the treatment effect for participants exposed to the drug for *up to* 18 months, while the latter concerns those exposed for the *full* 18 months. Global approaches assess treatment effects more realistically, mirroring real‐world usage, while single time point approaches evaluate under ideal conditions of consistent 18‐month treatment. The choice of method hinges first on the clinical question being addressed. These methods also differ in their reference disease progression trajectory for estimating time savings: BPP, ATNRP‐P, and PGP reference the placebo progression; BPT, ATNRP‐T, and PGT, the treatment progression. The former estimates time savings assuming discontinued active treatment, gauging how many more months the original treatment arm would take to match the placebo's end‐of‐study decline. The latter assumes ongoing active treatment, similar to an OLE phase,^1^, ^15^ estimating how many more months it would take for the original treatment arm to reach the observed end‐of‐study placebo decline. Given the typical continuation of active treatment during the OLE phase, estimating time savings relative to the treatment progression trajectory seems more practical. Therefore, the second factor to consider when selecting one method over another depends on the chosen reference of the disease progression trajectory.

Taking everything into account, we suggest giving priority to the global approaches, followed by the ATNRP methods, and last, BPT and BPP. One method could be designated as the primary model, with the others acting as sensitivity analyses. However, using multiple models is bound to produce some inconsistencies, which can be justified or explained by the distinct assumptions and the varying amounts of information used by each method. Considering that the direct outcome of many methods, whether ours or Raket's, is a proportional treatment effect, and that regardless of the metrics reported (be it time savings or absolute differences), they all tend to yield equivalent or closely aligned proportional treatment effects, thus, we advocate for assessing model performance consistency through the comparison of proportional treatment effects. Exceptions occur in scenarios in which the decline in the placebo arm not only diminishes over time but also equals (Figure 3B, Figure 6A) or becomes smaller than (Figure 3A, Figure 6B) that of the treatment arm, suggesting the treatment's efficacy is merely symptomatic or even inferior to placebo. In such cases, the time savings proportion estimated by BPP may significantly diverge from the proportion of disease progression reduction on the *y* axis. These situations may stem from differential dropout rates between the placebo and treatment groups, attributable to data not missing at random, as detailed in Section 2.2.1. They may also stem from non‐linear decline and the characteristics of the illness, outcome scales, learning effects, and enhanced clinical care that patients received in the trial.^3^, ^16^ They could lead to longer estimated time savings using the BPP method and shorter estimates with other methods. Given the difficulty in predicting how differential dropout rates or data missing not at random might influence disease progression patterns and, consequently, the estimated time savings, we reiterate our recommendation to prioritize global approaches. These methods offer a more comprehensive estimation over the entire trial duration, thereby minimizing the impact of differential dropout rates in the trial's later stages.

As OLE are customary extensions for phase 2 and phase 3 AD trials,^1^, ^10^, ^15^ the combination of the double‐blind, placebo‐controlled period with the OLE period forms a trial with a delayed‐start design.^17^, ^18^ Although all methods primarily aim to estimate time savings during the double‐blind, placebo‐controlled period, they also can be adapted to the delayed‐start context. This can allow for a comparison of time savings at the end of the placebo‐controlled period against those at the conclusion of the delayed‐start OLE period. When the change in CDR‐SB or any primary endpoint for both the placebo‐controlled and OLE periods is calculated from a single baseline, like the placebo‐controlled baseline, all proposed methods can be applied by incorporating data from both the placebo‐controlled and the OLE periods into the model. This approach allows for the initial estimation of the means changes, which serves as the basis for BPP, ATNRP‐P, BPT, and ATNRP‐T, or the proportional treatment effect for PGP and PGT. Subsequently, time savings for each period are calculated. Future work can evaluate the performance of each method within the framework of a delayed‐start design.

Our methodology has several limitations worth noting. Our approaches were exclusively applied to simulated trial data, meaning that the results presented in this study may not precisely mirror what could have been obtained using actual trial data especially with regard to assumptions on missingness. Second, while our key assumption, which suggests that the rates of disease progression before and after 18 months are comparable, is testable within our methods, there is currently a lack of long‐term real trial data for any disease‐modifying treatment to validate this assumption. Consequently, any results derived from the application of these approaches should be interpreted with caution. Third, although these new methods address the increased dropout rates over time, like BPP, no methods have addressed the unequal dropout rates between the treatment arms. Fourth, we did not directly compare our methods to Raket's due to the significant differences between our respective approaches. Instead, we conducted an indirect comparison between our PGP method and Raket's proportional time‐slowing method. This was achieved by applying the PGP method to simulate semi‐real trial CDR‐SB data for the low–medium tau population. Our estimated time savings, derived from pMMRM, amounted to 7.54 (95% confidence interval [CI], 5.50–9.57), while the estimate obtained using Raket's method,^3^ based on actual trial CDR‐SB data, was 7.53 months (95% CI, 5.69–9.36). Figure S5 and Table S9 in supporting information demonstrate the consistency in the estimated disease progression trajectories between the MMRM and pMMRM methods using the semi‐real trial data. Moreover, both our global approaches and those proposed by Raket hinge on the calculated proportional treatment effect to estimate time savings. Upcoming studies might explore using the area under the curve to calculate the differential area between the placebo and treatment arm curves, thereby estimating time savings. This methodology imposes no constraints on the disease progression trajectories, potentially offering broader applicability. Finally, while this study concentrates on calculating time savings through a univariate method centered on one endpoint, forthcoming research could adopt a multivariate strategy to estimate time savings across multiple endpoints, as previously detailed.^9^

Converting the treatment effect from an absolute difference in the primary endpoint (e.g., CDR‐SB) to time savings in disease progression can enhance interpretability. This approach makes it considerably simpler to convey how long the treatment has delayed disease progression in terms of time, as opposed to explaining a difference in a numerical scale that might not be readily understood by the general audience. Our approaches rely on fewer, yet testable, assumptions and can generate time‐saving treatment effects that accurately capture the variations in absolute differences at the end of the study. As a result, they can serve as valuable alternative tools for quantifying time savings in clinical trials for AD.

## CONFLICT OF INTEREST STATEMENT

Guoqiao Wang, PhD, is the biostatistics core co‐leader for the DIAN‐TU. He reports serving on a Data Safety Committee for Eli Lilly and Company and as a statistical consultant for Alector. Gary Cutter, PhD, reports serving on a Data Safety Committee and as a statistical consultant for many companies and the full list is available in the disclosure form. He also receives grant support from NIH. Neil P. Oxtoby, PhD, reports serving as a statistical consultant for Therapanacea (FR) and Queen Square Analytics Limited (UK). He also receives grant support from UKRI Medical Research Council. Jorge J. Llibre‐Guerra, PhD, receives support from the following grants: K01AG073526, SG‐20‐690363, AARFD‐21‐851415. Chengjie Xiong, PhD, reports serving as a statistical consultant for Diadem and for FDA Advisory Committee on Imaging Medical Products. He also receives support from the following grants: NIH Grant AG067505. Eric McDade, DO, is the Associate Director of the DIAN‐TU. He reports serving on a Data Safety Committee for Eli Lilly and Company and Alector; scientific consultant for Eisai and Eli Lilly and Company; institutional grant support from Eli Lilly and Company, F. Hoffmann‐La Roche, Ltd., and Janssen. Paul Delmar, PhD, is a stockholder of F. Hoffmann—La Roche Ltd company. Randall J. Bateman, MD, is the Director of the DIAN‐TU and Principal Investigator of the DIAN‐TU‐001. He receives research support from the National Institute on Aging of the National Institutes of Health, DIAN‐TU Trial Pharmaceutical Partners (Eli Lilly and Company, F. Hoffman‐La Roche, Ltd., and Avid Radiopharmaceuticals), Alzheimer's Association, GHR Foundation, Anonymous Organization, DIAN‐TU Pharma Consortium (Active: Biogen, Eisai, Eli Lilly and Company, Janssen, F. Hoffmann‐La Roche, Ltd./Genentech. Previous: AbbVie, Amgen, AstraZeneca, Forum, Mithridion, Novartis, Pfizer, Sanofi, United Neuroscience). He has been an invited speaker for Novartis and serves on the Advisory Board for F. Hoffman La Roche, Ltd. Lon Schneider, MD, reports serving on a Data Safety Committee and as a statistical consultant for many companies and the full list is available in the disclosure form. He also receives grant support from NIH. All the other authors reported no conflicts of interest for this study. Author disclosures are available in the Supporting information.

## CONSENT STATEMENT

Informed consent from all human subjects was not necessary.

## Supporting information

Supporting Information

Supporting Information
