# Supplementary material for: Statistical considerations when estimating time‐saving treatment effects in Alzheimer's disease clinical trials
Source: Alzheimers Dement. 2024 Jun 21;20(8):5421–33. doi: 10.1002/alz.14035 (PMC11350030; doi:10.1002/alz.14035)
Supplement: Supplementary file 1 — Supporting Information [file ALZ-20-5421-s001.docx]

**Supplemental Table 1**: Data used to generate Figure 3

|  | **3A*** | | | | **3B*** | | | | **3C****‡** | | | | **3D‡** | | | |
| --- | --- | --- | --- | --- | --- | --- | --- | --- | --- | --- | --- | --- | --- | --- | --- | --- |
|  | **Mean change**  **from baseline** | | **6-month decline** | | **Mean change**  **from baseline** | | **6-month decline** | | **Mean change**  **from baseline** | | **6-month decline** | | **Mean change**  **from baseline** | | **6-month decline** | |
| **Month** | **Placebo** | **Active** | **Placebo** | **Active** | **Placebo** | **Active** | **Placebo** | **Active** | **Placebo** | **Active** | **Placebo** | **Active** | **Placebo** | **Active** | **Placebo** | **Active** |
| 0 | 0.0 | 0.0 | NA | NA | 0.0 | 0.0 | NA | NA | 0.0 | 0.0 | NA | NA | 0.0 | 0.0 | NA | NA |
| 6 | 0.6 | 0.5 | NA | NA | 0.6 | 0.5 | NA | NA | 0.6 | 0.5 | NA | NA | 0.6 | 0.5 | NA | NA |
| 12 | 1.6 | 1.1 | 1.0 | 0.6 | 1.6 | 1.1 | 1.0 | 0.6 | 1.6 | 1.1 | 1.0 | 0.6 | 1.6 | 1.1 | 1.0 | 0.6 |
| 18 | 2.2 | 1.7 | 0.6 | 0.6 | 2.2 | 1.7 | 0.6 | 0.6 | 2.2 | 1.7 | 0.6 | 0.6 | 2.2 | 1.7 | 0.6 | 0.6 |
| 24 | 2.6 | 2.1 | 0.4 | 0.4 | 2.6 | 2.1 | 0.4 | 0.4 | 2.8 | 2.3 | 0.6 | 0.6 | 2.9 | 2.4 | 0.7 | 0.7 |
| 30 | 2.9 | 2.5 | 0.3 | 0.4 | 3.0 | 2.5 | 0.4 | 0.4 | 3.4 | 2.9 | 0.6 | 0.6 | 3.7 | 3.2 | 0.8 | 0.8 |
| 36 | 3.1 | 2.8 | 0.2 | 0.3 | 3.3 | 2.8 | 0.3 | 0.3 | 4.0 | 3.5 | 0.6 | 0.6 | 4.6 | 4.1 | 0.9 | 0.9 |
| *3A was recreated based on Racket’s MMRM Figure^1^, and 3B was adapted from Petersen et al.^2^. The values such as the mean change from baseline to each visit were approximately extracted from their respective figures, thus may be slightly different from those used in their figures.  ‡3C and 3D are adapted based on 3A and 3B.  6-month decline is calculated by taking the different between two consecutive visits starting from month 6. | | | | | | | | | | | | | | | | |

**Supplemental Table 2**: Mean change from baseline and the visit-interval declines in CDR SB

| **Month** | **Mean change from baseline*** | | **3-month decline****ǂ** | | **Week** | **Mean change from baseline*** | | **~12-week decline ǂ** | |
| --- | --- | --- | --- | --- | --- | --- | --- | --- | --- |
|  | **Placebo** | **Lecanemab~** | **Placebo** | **Lecanemab** |  | **Placebo** | **Donanemab^ǁ^** | **Placebo** | **Donanemab** |
| 3 | 0.36 | 0.27 | NA | NA | 12 | 0.28 | 0.18 | NA | NA |
| 6 | 0.61 | 0.43 | 0.25 | 0.16 | 24 | 0.64 | 0.33 | 0.36 | 0.15 |
| 9 | 0.80 | 0.58 | 0.19 | 0.15 | 36 | 0.95 | 0.46 | 0.31 | 0.13 |
| 12 | 1.16 | 0.81 | 0.36 | 0.23 | 52 | 1.35 | 0.75 | 0.40 | 0.29 |
| 15 | 1.41 | 1.02 | 0.25 | 0.21 | 64 | 1.53 | 0.94 | 0.18 | 0.19 |
| 18 | 1.66 | 1.21 | 0.25 | 0.19 | 76 | 1.88 | 1.20 | 0.35 | 0.26 |
| * Mean values have been extracted from published figures for all visits except the last one. As a result, they approximate rather than accurately represent the true values estimated from actual trial data.  ǂ Calculated as the difference between two consecutive follow-up visits, e.g., 0.25=0.61-0.36.  NA: not applicable  ~Clarity AD lecanemab trial^3^  ǁTRAILBLAZER‐ALZ 2 donanemab trial (low/median tau population)^4^ | | | | | | | | | |

**Supplemental Table** **3**: Mean changes from baseline and sample sizes of the three simulated clinical trials

| **Month** | **Placebo sample size** | **Simulated lecanemab**  **sample size** | **Difference in Sample Size between arms** | **Placebo** | **Simulated lecanemab** | **Decelerated placebo** | **Accelerated placebo** |
| --- | --- | --- | --- | --- | --- | --- | --- |
| 0 | 875 | 859 | 16 | 0.00 | 0.00 | 0.00 | 0.00 |
| 3 | 849 | 824 | 25 | 0.36 | 0.27 | 0.36 | 0.36 |
| 6 | 828 | 798 | 30 | 0.61 | 0.43 | 0.61 | 0.61 |
| 9 | 813 | 779 | 34 | 0.80 | 0.58 | 0.80 | 0.80 |
| 12 | 779 | 765 | 14 | 1.16 | 0.81 | 1.16 | 1.16 |
| 15 | 767 | 738 | 29 | 1.41 | 1.02 | 1.41 | 1.41 |
| 18 | 757 | 714 | 43 | 1.66 | 1.21 | 1.40 | 1.84 |

**Supplemental Table** **4**: Estimated time savings (in months) for each method across various scenarios

|  | **Method** | **Estimate** | **SE** | **t Value** | **Pr > \|t\|** | **95% CI**  **Lower Bound** | **95% CI**  **Upper Bound** |
| --- | --- | --- | --- | --- | --- | --- | --- |
| **Figure 1A** | **BPP** | 5.40 | 1.18 | 4.59 | <.0001 | 3.09 | 7.71 |
|  | **BPT** | 6.65 | 1.96 | 3.39 | 0.0007 | 2.80 | 10.50 |
|  | **ATNRP-P** | 4.88 | 1.04 | 4.70 | <.0001 | 2.84 | 6.92 |
|  | **ATNRP-T** | 6.69 | 1.96 | 3.42 | 0.0006 | 2.86 | 10.53 |
|  | **PGP** | 5.02 | 0.95 | 5.28 | <.0001 | 3.16 | 6.89 |
|  | **PGT** | 6.97 | 1.83 | 3.81 | 0.0001 | 3.38 | 10.56 |
| **Figure 1B** | **BPP** | 5.40 | 1.18 | 4.59 | <.0001 | 3.09 | 7.71 |
|  | **BPT** | 3.00 | 2.11 | 1.42 | 0.155 | -1.14 | 7.14 |
|  | **ATNRP-P** | 2.44 | 1.31 | 1.86 | 0.0631 | -0.13 | 5.02 |
|  | **ATNRP-T** | 2.83 | 1.76 | 1.61 | 0.1081 | -0.62 | 6.27 |
|  | **PGP** | 4.23 | 1.06 | 4.00 | <.0001 | 2.15 | 6.30 |
|  | **PGT** | 5.52 | 1.80 | 3.06 | 0.0022 | 1.98 | 9.06 |
| **Figure 1C** | **BPP** | 5.40 | 1.18 | 4.59 | <.0001 | 3.09 | 7.71 |
|  | **BPT** | 9.00 | 2.08 | 4.33 | <.0001 | 4.93 | 13.07 |
|  | **ATNRP-P** | 6.16 | 0.91 | 6.79 | <.0001 | 4.38 | 7.94 |
|  | **ATNRP-T** | 9.37 | 2.10 | 4.47 | <.0001 | 5.26 | 13.49 |
|  | **PGP** | 5.95 | 0.87 | 6.86 | <.0001 | 4.25 | 7.65 |
|  | **PGT** | 8.89 | 1.94 | 4.59 | <.0001 | 5.09 | 12.69 |

**Supplemental Table** **5**: Estimated mean changes from baseline for each method across various scenarios

| Month | Scenario: Figure 1A | | | | Scenario: Figure 1B | | | | Scenario: Figure 1C | | | |
| --- | --- | --- | --- | --- | --- | --- | --- | --- | --- | --- | --- | --- |
|  | MMRM | | pMMRM | | MMRM | | pMMRM | | MMRM | | pMMRM | |
|  | **Placebo** | **Treatment** | **Placebo** | **Treatment** | **Placebo** | **Treatment** | **Placebo** | **Treatment** | **Placebo** | **Treatment** | **Placebo** | **Treatment** |
| 0 | 0 | 0 | 0 | 0 | 0 | 0 | 0 | 0 | 0 | 0 | 0 | 0 |
| 3 | 0.36 | 0.27 | 0.36 | 0.26 | 0.36 | 0.27 | 0.36 | 0.27 | 0.36 | 0.27 | 0.37 | 0.25 |
| 6 | 0.61 | 0.43 | 0.61 | 0.44 | 0.61 | 0.43 | 0.59 | 0.45 | 0.61 | 0.43 | 0.62 | 0.41 |
| 9 | 0.80 | 0.58 | 0.80 | 0.58 | 0.80 | 0.58 | 0.78 | 0.60 | 0.80 | 0.58 | 0.82 | 0.55 |
| 12 | 1.16 | 0.81 | 1.15 | 0.83 | 1.16 | 0.81 | 1.12 | 0.86 | 1.16 | 0.81 | 1.18 | 0.79 |
| 15 | 1.41 | 1.02 | 1.41 | 1.02 | 1.41 | 1.02 | 1.38 | 1.06 | 1.41 | 1.02 | 1.44 | 0.97 |
| 18 | 1.66 | 1.21 | 1.67 | 1.20 | 1.40 | **1.21** | 1.46 | **1.12** | 1.84 | 1.21 | 1.83 | 1.23 |
| pMMRM: proportional MMRM with the placebo disease progression as the reference  Largest discrepancy was observed when the treatment effect faded over time (Scenario: Figure 1B) | | | | | | | | | | | | |

**Supplemental Table 6**: Mean changes from baseline to each post-baseline visit in CDR SB for various treatment effects

|  | **Symptomatic improvement (constant)** | | | **Symptomatic improvement (fading)** | | | **Reducing decline (proportional)** | | |
| --- | --- | --- | --- | --- | --- | --- | --- | --- | --- |
| **Month** | **Placebo** | **Treatment** | **Difference** | **Placebo** | **Treatment** | **Difference** | **Placebo** | **Treatment** | **Difference** |
| 3 | 0.36 | 0.27 | 0.09 | 0.36 | 0.27 | 0.09 | 0.36 | 0.25 | 0.11 |
| 6 | 0.61 | 0.43 | 0.18 | 0.61 | 0.43 | 0.18 | 0.61 | 0.43 | 0.18 |
| 9 | 0.80 | 0.58 | 0.22 | 0.80 | 0.58 | 0.22 | 0.80 | 0.56 | 0.24 |
| 12 | 1.16 | 0.94 | 0.22 | 1.16 | 0.94 | 0.22 | 1.16 | 0.81 | 0.35 |
| 15 | 1.41 | 1.19 | 0.22 | 1.41 | 1.24 | 0.17 | 1.41 | 0.99 | 0.42 |
| 18 | 1.66 | 1.44 | 0.22 | 1.66 | 1.54 | 0.12 | 1.66 | 1.16 | 0.50 |
|  | **Delaying progression (constant)** | | | **Slowing progression (Proportional)** | | | **Slowing progression (non-structured)** | | |
| **Month** | **Placebo** | **Treatment** | **Difference** | **Placebo** | **Treatment** | **Difference** | **Placebo** | **Treatment** | **Difference** |
| 3 | 0.36 | 0.30 | 0.06 | 0.36 | 0.25 | 0.11 | 0.36 | 0.25 | 0.11 |
| 6 | 0.61 | 0.52 | 0.09 | 0.61 | 0.46 | 0.15 | 0.61 | 0.46 | 0.15 |
| 9 | 0.80 | 0.68 | 0.12 | 0.80 | 0.63 | 0.17 | 0.80 | 0.63 | 0.17 |
| 12 | 1.16 | 0.98 | 0.18 | 1.16 | 0.76 | 0.40 | 1.16 | 0.76 | 0.40 |
| 15 | 1.41 | 1.29 | 0.12 | 1.41 | 0.98 | 0.43 | 1.41 | 0.98 | 0.43 |
| 18 | 1.66 | 1.54 | 0.13 | 1.66 | 1.21 | 0.45 | 1.66 | 1.02 | 0.65 |
| **Difference:** difference between placebo and treatment in the mean change from baseline | | | | | | | | | |

**Supplemental Table 7**: Estimated time savings for each method across various scenarios

|  | **Method** | **Estimate** | **SE** | **t Value** | **Pr > \|t\|** | **95% CI**  **Lower Bound** | **95% CI**  **Upper Bound** |
| --- | --- | --- | --- | --- | --- | --- | --- |
| **Symptomatic**  **improvement**  **(constant)** | **BPP** | 2.63 | 1.24 | 2.12 | 0.0345 | 0.19 | 5.07 |
|  | **BPT** | 2.63 | 1.56 | 1.69 | 0.0915 | -0.43 | 5.68 |
|  | **ATNRP-P** | 2.38 | 1.11 | 2.14 | 0.0324 | 0.20 | 4.55 |
|  | **ATNRP-T** | 2.74 | 1.47 | 1.86 | 0.0633 | -0.15 | 5.63 |
|  | **PGP** | 2.80 | 1.01 | 2.77 | 0.0057 | 0.81 | 4.79 |
|  | **PGT** | 3.32 | 1.42 | 2.34 | 0.0196 | 0.53 | 6.10 |
| **Symptomatic**  **improvement**  **(fading)** | **BPP** | 1.43 | 1.25 | 1.14 | 0.2528 | -1.02 | 3.88 |
|  | **BPT** | 1.19 | 1.17 | 1.02 | 0.309 | -1.10 | 3.49 |
|  | **ATNRP-P** | 1.29 | 1.14 | 1.13 | 0.2586 | -0.95 | 3.53 |
|  | **ATNRP-T** | 1.39 | 1.33 | 1.05 | 0.2943 | -1.21 | 3.99 |
|  | **PGP** | 2.03 | 1.04 | 1.95 | 0.0512 | -0.01 | 4.06 |
|  | **PGT** | 2.28 | 1.32 | 1.73 | 0.0836 | -0.30 | 4.87 |
| **Reducing decline**  **(proportional)** | **BPP** | 5.98 | 1.18 | 5.06 | <.0001 | 3.66 | 8.29 |
|  | **BPT** | 7.76 | 1.83 | 4.23 | <.0001 | 4.17 | 11.36 |
|  | **ATNRP-P** | 5.40 | 1.03 | 5.27 | <.0001 | 3.39 | 7.41 |
|  | **ATNRP-T** | 7.71 | 2.09 | 3.69 | 0.0002 | 3.61 | 11.82 |
|  | **PGP** | 5.40 | 0.94 | 5.74 | <.0001 | 3.56 | 7.25 |
|  | **PGT** | 7.71 | 1.92 | 4.02 | <.0001 | 3.95 | 11.48 |
|  | **BPP** | 1.50 | 1.25 | 1.20 | 0.2294 | -0.95 | 3.95 |
| **Delaying**  **progression**  **(constant)** | **BPT** | 1.50 | 1.43 | 1.05 | 0.2957 | -1.31 | 4.31 |
|  | **ATNRP-P** | 1.36 | 1.14 | 1.19 | 0.2349 | -0.88 | 3.59 |
|  | **ATNRP-T** | 1.47 | 1.33 | 1.10 | 0.272 | -1.15 | 4.08 |
|  | **PGP** | 1.80 | 1.04 | 1.74 | 0.0828 | -0.23 | 3.84 |
|  | **PGT** | 2.00 | 1.28 | 1.56 | 0.1184 | -0.51 | 4.52 |
| **Slowing**  **progression**  **(Proportional)** | **BPP** | 5.40 | 1.18 | 4.59 | <.0001 | 3.09 | 7.71 |
|  | **BPT** | 6.04 | 3.37 | 1.80 | 0.0727 | -0.56 | 12.64 |
|  | **ATNRP-P** | 4.88 | 1.04 | 4.70 | <.0001 | 2.84 | 6.92 |
|  | **ATNRP-T** | 6.69 | 1.96 | 3.42 | 0.0006 | 2.86 | 10.53 |
|  | **PGP** | 5.13 | 0.96 | 5.35 | <.0001 | 3.25 | 7.01 |
|  | **PGT** | 7.18 | 1.88 | 3.83 | 0.0001 | 3.50 | 10.86 |
| **Slowing**  **progression**  **(non-structured)** | **BPP** | 7.20 | 0.79 | 9.13 | <.0001 | 5.65 | 8.75 |
|  | **BPT** | 13.27 | 2.41 | 5.51 | <.0001 | 8.55 | 17.99 |
|  | **ATNRP-P** | 6.98 | 0.99 | 7.08 | <.0001 | 5.05 | 8.92 |
|  | **ATNRP-T** | 11.41 | 2.63 | 4.34 | <.0001 | 6.25 | 16.57 |
|  | **PGP** | 6.29 | 0.93 | 6.73 | <.0001 | 4.46 | 8.13 |
|  | **PGT** | 9.67 | 2.21 | 4.38 | <.0001 | 5.34 | 14.01 |

**Supplemental Table 8**: Mean changes from baseline to each post-baseline visit in CDR SB estimated by MMRM and pMMRM

|  | **Symptomatic improvement (constant)** | | | | **Symptomatic improvement (fading)** | | | | **Reducing decline (proportional)** | | | |
| --- | --- | --- | --- | --- | --- | --- | --- | --- | --- | --- | --- | --- |
|  | **MMRM** | | **pMMRM** | | **MMRM** | | **pMMRM** | | **MMRM** | | **pMMRM** | |
| Month | Placebo | Treatment | Placebo | Treatment | Placebo | Treatment | Placebo | Treatment | Placebo | Treatment | Placebo | Treatment |
| 0 | 0 | 0 | 0 | 0 | 0 | 0 | 0 | 0 | 0 | 0 | 0 | 0 |
| 3 | 0.360 | 0.270 | 0.344 | 0.290 | 0.360 | 0.270 | 0.336 | 0.298 | 0.360 | 0.252 | 0.360 | 0.252 |
| 6 | 0.610 | 0.430 | 0.569 | 0.480 | 0.610 | 0.430 | 0.556 | 0.493 | 0.610 | 0.427 | 0.610 | 0.427 |
| 9 | 0.800 | 0.580 | 0.754 | 0.637 | 0.800 | 0.580 | 0.737 | 0.654 | 0.800 | 0.560 | 0.800 | 0.560 |
| 12 | 1.160 | 0.941 | 1.142 | 0.964 | 1.160 | 0.941 | 1.117 | 0.991 | 1.160 | 0.812 | 1.160 | 0.812 |
| 15 | 1.410 | 1.191 | 1.410 | 1.191 | 1.410 | 1.241 | 1.405 | 1.247 | 1.410 | 0.987 | 1.410 | 0.987 |
| 18 | 1.660 | 1.441 | 1.679 | 1.418 | 1.660 | 1.541 | 1.693 | 1.502 | 1.660 | 1.162 | 1.660 | 1.162 |
|  | **Delaying progression (constant)** | | | | **Slowing progression (Proportional)** | | | | **Slowing progression (non-structured)** | | | |
|  | **MMRM** | | **pMMRM** | | **MMRM** | | **pMMRM** | | **MMRM** | | **pMMRM** | |
| Month | Placebo | Treatment | Placebo | Treatment | Placebo | Treatment | Placebo | Treatment | Placebo | Treatment | Placebo | Treatment |
| 0 | 0 | 0 | 0 | 0 | 0 | 0 | 0 | 0 | 0 | 0 | 0 | 0 |
| 3 | 0.360 | 0.300 | 0.348 | 0.313 | 0.360 | 0.252 | 0.358 | 0.256 | 0.360 | 0.252 | 0.368 | 0.239 |
| 6 | 0.610 | 0.520 | 0.596 | 0.536 | 0.610 | 0.460 | 0.621 | 0.444 | 0.610 | 0.460 | 0.638 | 0.415 |
| 9 | 0.800 | 0.680 | 0.781 | 0.702 | 0.800 | 0.629 | 0.826 | 0.591 | 0.800 | 0.629 | 0.848 | 0.552 |
| 12 | 1.160 | 0.980 | 1.129 | 1.016 | 1.160 | 0.762 | 1.128 | 0.807 | 1.160 | 0.762 | 1.163 | 0.756 |
| 15 | 1.410 | 1.285 | 1.418 | 1.276 | 1.410 | 0.980 | 1.396 | 0.998 | 1.410 | 0.980 | 1.437 | 0.935 |
| 18 | 1.660 | 1.535 | 1.680 | 1.512 | 1.660 | 1.210 | 1.670 | 1.194 | 1.660 | 1.016 | 1.632 | 1.061 |

**Supplemental Table 9**: Mean change from baseline to each post-baseline visit in CDR SB estimated by MMRM and pMMRM for the low-medium tau population based on simulated semi-real trial data.

|  | **MMRM** | | **pMMRM** | |
| --- | --- | --- | --- | --- |
| **Weeks** | **Placebo** | **Treatment** | **Placebo** | **Treatment** |
| 0 | 0 | 0 | 0 | 0 |
| 12 | 0.283 | 0.180 | 0.287 | 0.173 |
| 24 | 0.640 | 0.332 | 0.618 | 0.373 |
| 36 | 0.945 | 0.460 | 0.899 | 0.543 |
| 52 | 1.345 | 0.754 | 1.321 | 0.797 |
| 64 | 1.526 | 0.941 | 1.535 | 0.926 |
| 76 | 1.880 | 1.200 | 1.908 | 1.151 |


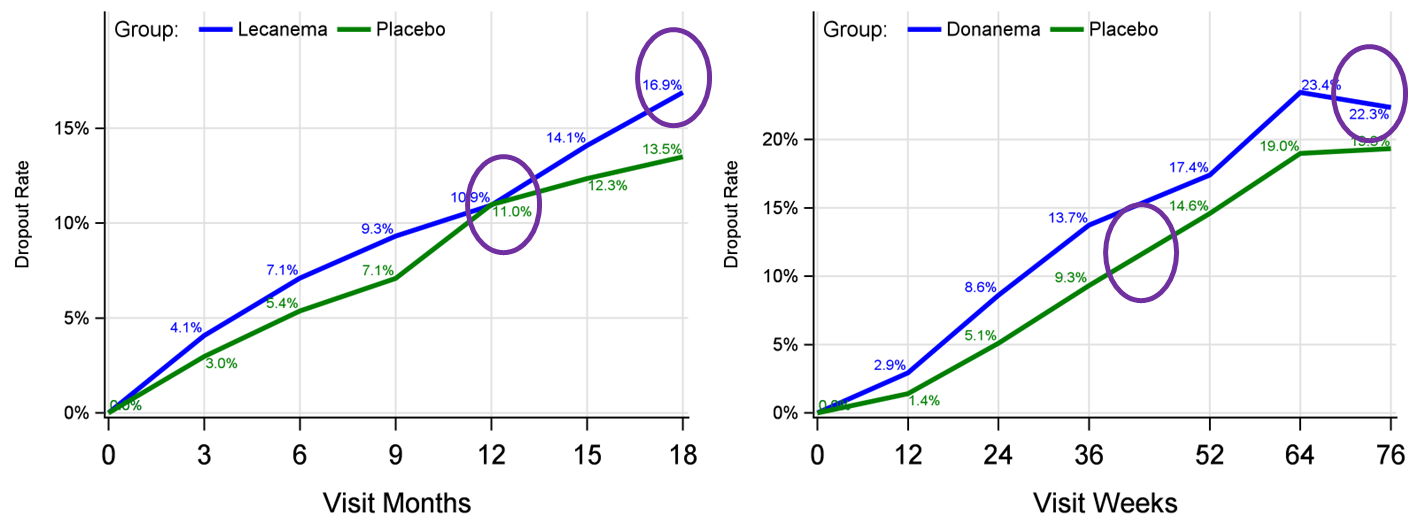


**Supplemental Figure 1**: Dropout rates for lecanemab (Left) and donanemab (right) trials (low/median tau population). (Dropout rates were calculated from van Dyck et al.^5^ and Sims et al.^4^).


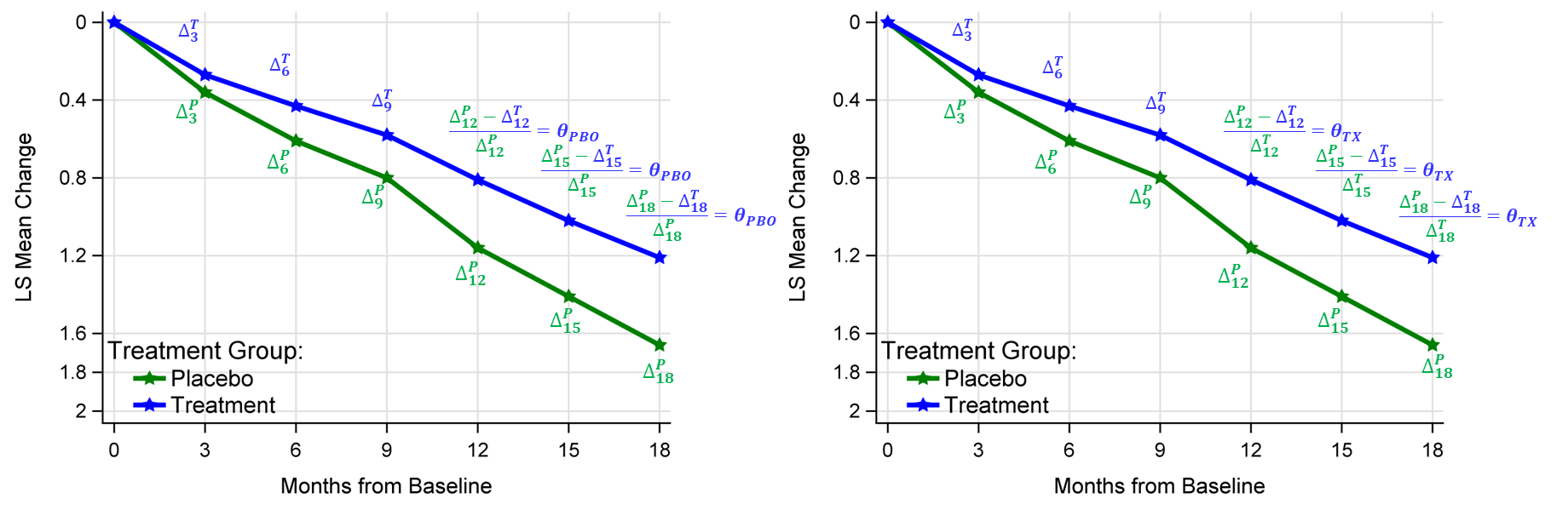


**Supplemental Figure 2**: Demonstration of estimating the proportional treatment effect using multiple (months 12 to 18) but not all post-baseline visits.


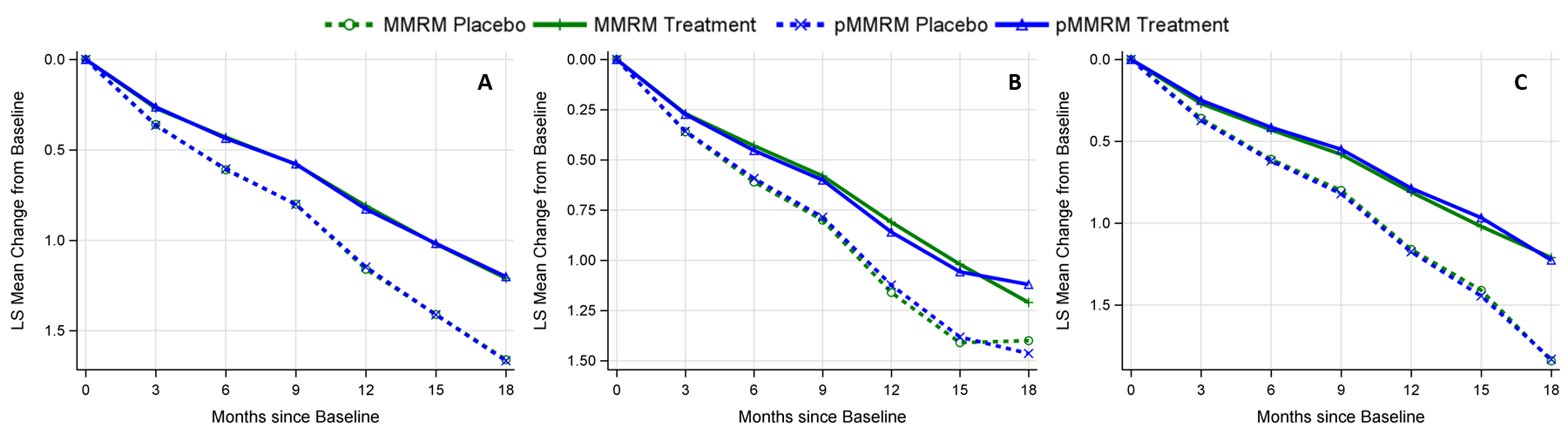


**Supplemental Figure 3**: Demonstration of the estimated disease progression trajectories using MMRM and pMMRM methods. Despite the use of a proportional treatment effect, pMMRM demonstrates strong consistency with MMRM's trajectories. Notable deviation occurred at the 18-month mark when the treatment effect diminished (panel B), with a discrepancy of 0.09 in the progression trajectories of the treatment groups. Both MMRM and pMMRM were applied to simulated semi-real trial data. Using the MMRM analysis, the semi-real trial data allows the reproduction of the corresponding published figure generated using the real trial data. MMRM: mixed model for repeated measures; pMMRM: proportional MMRM.


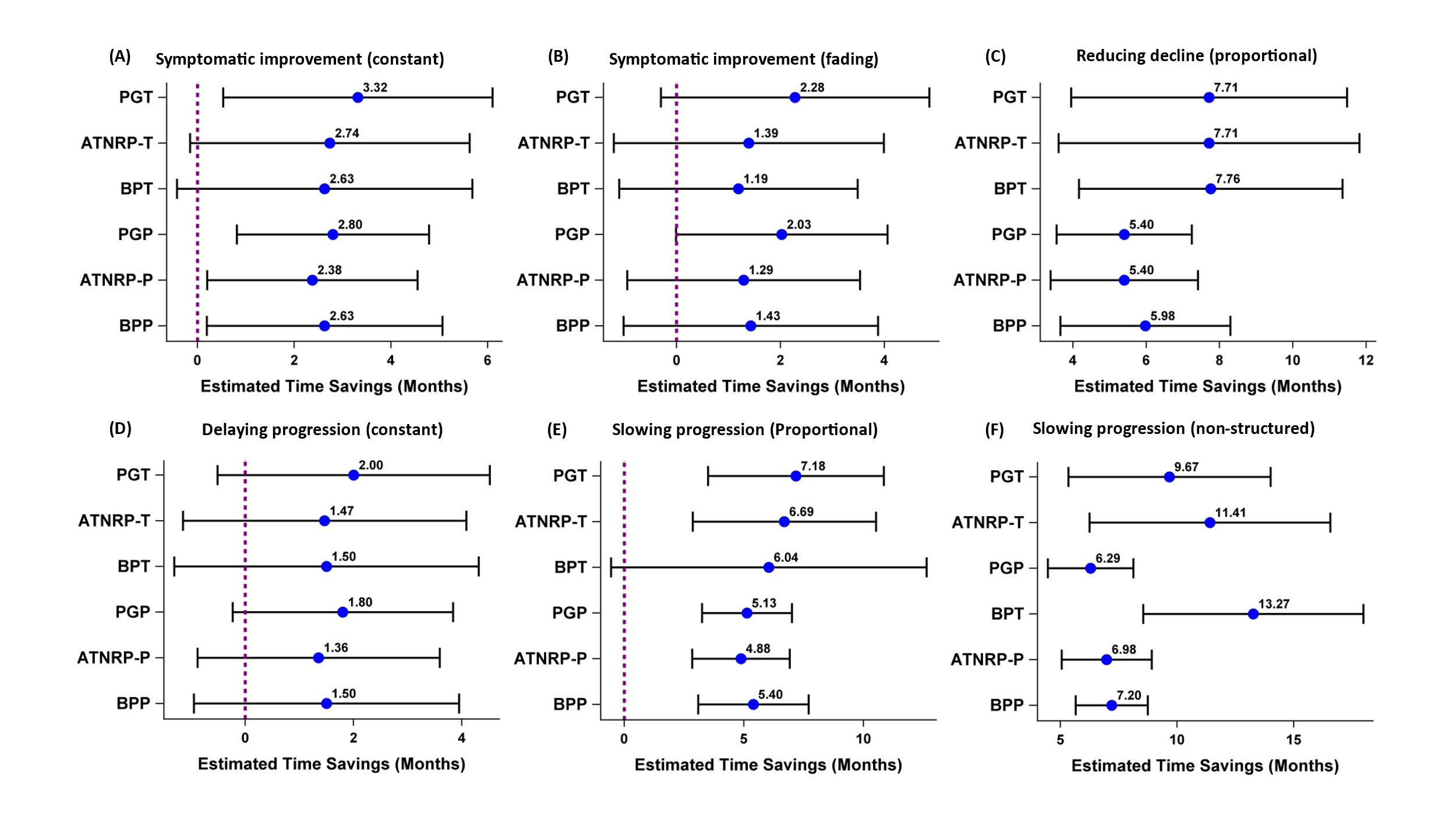


**Supplemental Figure 4**: Demonstration of estimated time savings and the 95% confidence intervals across methods and disease progression patterns. BPP: Backward projection to the placebo decline; BPT: Backward projection to the treatment decline; ATNRP-P: Additional time needed to reach the placebo decline relative to the disease progression rate observed in the placebo arm; ATNRP-T: Additional time needed to reach the placebo decline relative to the disease progression rate observed in the treatment arm; PGP: Proportional global time savings relative to placebo; PGT: Proportional global time savings relative to treatment.


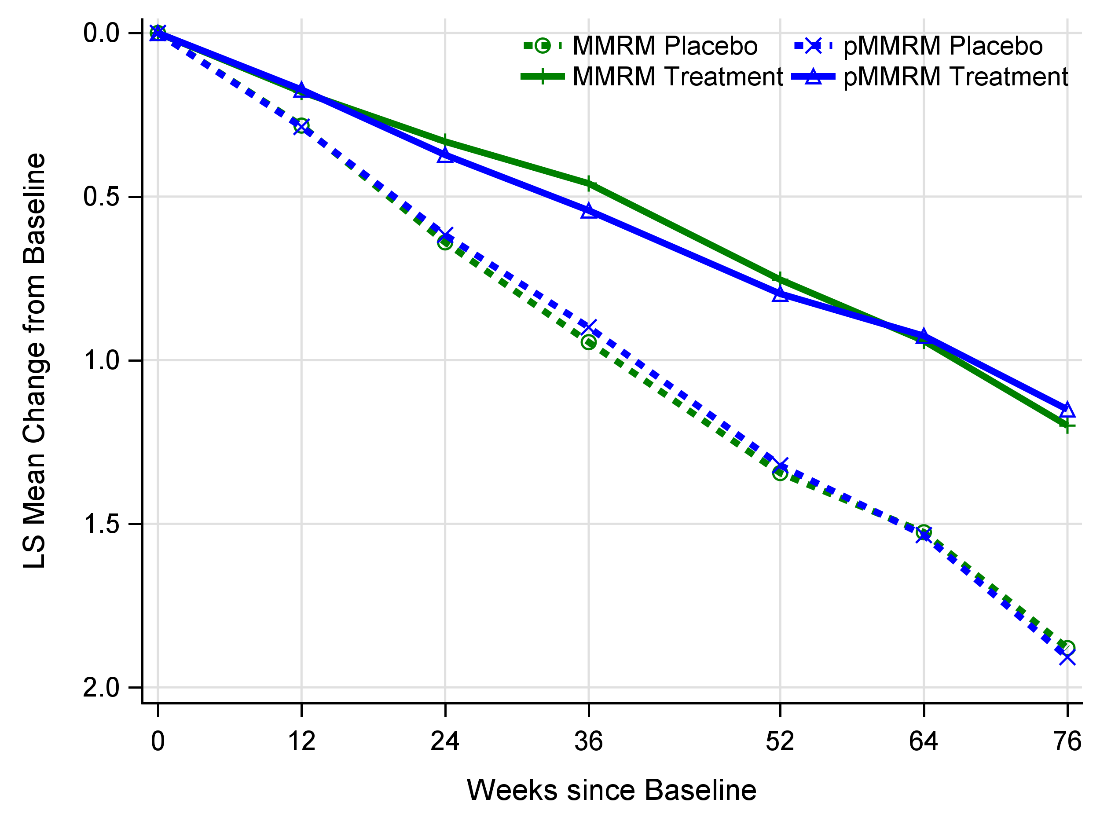


**Supplemental Figure 5**: Illustration of consistency between disease progression trajectories estimated by MMRM and pMMRM for the low-medium tau Population. The estimated proportional treatment effect is 39.7% (29.0%, 50.4%), leading to an estimated time savings of 7.54 (95% CI, 5.50-9.57). Racket’s method^4^, based on actual trial CDR SB data, yielded an estimate of 7.53 months (95% CI, 5.69-9.36). Both MMRM and pMMRM were applied to simulated semi-real trial data. Using the MMRM analysis, the semi-real trial data allows the reproduction of the corresponding published figure generated using the real trial data. Despite the use of a proportional treatment effect, pMMRM demonstrates strong consistency with MMRM's trajectories. MMRM: mixed model for repeated measures; pMMRM: proportional MMRM.

1. Raket LL. A new approach for estimating and quantifying disease‐modification in Alzheimer’s disease clinical trials. *Alzheimer's & Dementia.* 2022;18:e063092.

2. Petersen RC, Aisen PS, Andrews JS, et al. Expectations and clinical meaningfulness of randomized controlled trials. *Alzheimer's & Dementia.* 2023.

3. Van Dyck CH, Swanson CJ, Aisen P, et al. Lecanemab in early Alzheimer’s disease. *New England Journal of Medicine.* 2023;388(1):9-21.

4. Sims JR, Zimmer JA, Evans CD, et al. Donanemab in early symptomatic Alzheimer disease: the TRAILBLAZER-ALZ 2 randomized clinical trial. *JAMA.* 2023.

5. van Dyck CH, Swanson CJ, Aisen P, et al. Lecanemab in early Alzheimer’s disease. *New England Journal of Medicine.* 2022.
