# Supplementary material for: Statistical considerations when estimating time‐saving treatment effects in Alzheimer's disease clinical trials
Source: Alzheimers Dement. 2024 Jun 21;20(8):5421–33. doi: 10.1002/alz.14035 (PMC11350030; doi:10.1002/alz.14035)
Supplement: Supplementary file 2 — Supporting Information [file ALZ-20-5421-s002.pdf]

ICMJE DISCLOSURE FORM

Date: 4/18/2024

Your Name: Guoqiao Wang

Manuscript Title: **Examining Amyloid Reduction as A Surrogate Endpoint through Latent Class Analysis Using Clinical Trial Data for Dominantly Inherited Alzheimer's Disease**

Manuscript Number (if known): ADJ-D-24-00175

In the interest of transparency, we ask you to disclose all relationships/activities/interests listed below that are related to the content of your manuscript. "Related" means any relation with for-profit or not-for-profit third parties whose interests may be affected by the content of the manuscript. Disclosure represents a commitment to transparency and does not necessarily indicate a bias. If you are in doubt about whether to list a relationship/activity/interest, it is preferable that you do so.

The author's relationships/activities/interests should be defined broadly. For example, if your manuscript pertains to the epidemiology of hypertension, you should declare all relationships with manufacturers of antihypertensive medication, even if that medication is not mentioned in the manuscript.

In item #1 below, report all support for the work reported in this manuscript without time limit. For all other items, the time frame for disclosure is the past 36 months.

		Name all entities with whom you have this relationship or indicate none (add rows as needed)	Specifications/Comments (e.g., if payments were made to you or to your institution)
**Time frame: Since the initial planning of the work**			
1	All support for the present manuscript (e.g., funding, provision of study materials, medical writing, article processing charges, etc.) **No time limit for this item.**	☐ **None**	
		NIH grant for DIAN TU	Grants to institution
			Click the tab key to add additional rows.
**Time frame: past 36 months**			
**2**	Grants or contracts from any entity (if not indicated in item #1 above).	☒ **None**	
**3**	Royalties or licenses	☒ **None**	

		Name all entities with whom you have this relationship or indicate none (add rows as needed)	Specifications/Comments (e.g., if payments were made to you or to your institution)
**4**	Consulting fees	☐ **None**	
		Alector	Payment to me
**5**	Payment or honoraria for lectures, presentations, speakers bureaus, manuscript writing or educational events	☒ **None**	
**6**	Payment for expert testimony	☒ **None**	
**7**	Support for attending meetings and/or travel	☒ **None**	
**8**	Patents planned, issued or pending	☒ **None**	
**9**	Participation on a Data Safety Monitoring Board or Advisory Board	☐ **None**	
		Eli Lilly	Payments to me
**10**	Leadership or fiduciary role in other board, society, committee or advocacy group, paid or unpaid	☒ **None**	

		Name all entities with whom you have this relationship or indicate none (add rows as needed)	Specifications/Comments (e.g., if payments were made to you or to your institution)
**11**	Stock or stock options	☒ **None**	
**12**	Receipt of equipment, materials, drugs, medical writing, gifts or other services	☒ **None**	
**13**	Other financial or non-financial interests	☒ **None**	

Please place an "X" next to the following statement to indicate your agreement:

☒ I certify that I have answered every question and have not altered the wording of any of the questions on this form.

ICMJE DISCLOSURE FORM

Date: 4/7/2024

Your Name: Gary Cutter, PhD

Manuscript Title: Statistical Considerations When Estimating Time-Saving Treatment Effects in Alzheimer's Clinical Trials

Manuscript Number (if known): ADJ-D-24-00175

In the interest of transparency, we ask you to disclose all relationships/activities/interests listed below that are related to the content of your manuscript. "Related" means any relation with for-profit or not-for-profit third parties whose interests may be affected by the content of the manuscript. Disclosure represents a commitment to transparency and does not necessarily indicate a bias. If you are in doubt about whether to list a relationship/activity/interest, it is preferable that you do so.

The author's relationships/activities/interests should be defined broadly. For example, if your manuscript pertains to the epidemiology of hypertension, you should declare all relationships with manufacturers of antihypertensive medication, even if that medication is not mentioned in the manuscript.

In item #1 below, report all support for the work reported in this manuscript without time limit. For all other items, the time frame for disclosure is the past 36 months.

		**Name all entities with whom you have this relationship or indicate none (add rows as needed)**	**Specifications/Comments (e.g., if payments were made to you or to your institution)**
		**Time frame: Since the initial planning of the work**	
1	All support for the present manuscript (e.g., funding, provision of study materials, medical writing, article processing charges, etc.) **No time limit for this item.**	☒ None	
			Click the tab key to add additional rows.
		**Time frame: past 36 months**	
2	Grants or contracts from any entity (if not indicated in item #1 above).	☐ **None**	
		Transferring Speed of Processing Gains to Everyday Cognitive Tasks after Stroke	R01AG070049
		Rare Disease Network for Myasthenia Gravis	U54 NS115054
		The Exercise and Physical Activity Collaborative Team (ExPACT): A MoTrPAC Clinical Center	2U01AR071133
		Host Immune Responses to Chlamydia trachomatis Candidate Vaccine Antigens and their Association with Clinical Correlates of Protective Immunity in Women	R01 AI148359
		Pregnancy as a Window to the Future: Outcomes of Antihypertensive Therapy and Superimposed Preeclampsia in Pregnant Women with Mild	R01 HL120338

		Name all entities with whom you have this relationship or indicate none (add rows as needed)	Specifications/Comments (e.g., if payments were made to you or to your institution)
		Chronic Hypertension (CHAP Maternal Follow-Up Study)	
		Video Telehealth Pulmonary Rehabilitation to Reduce Hospital Readmission in Chronic Obstructive Pulmonary Disease (Tele-COPD)	U24 HL155807
		Premature Infants Receiving Cord Milking or Delayed Cord Clamping	R01 HD088646
		Technique to Enable Return-to-Work by Employees with Long COVID Brain Fog	90IFRE0073
		UAB-UCSD O'Brien Center for Acute Kidney Injury Research	U54 DK137307
		Deep South KUH Premier Research and Inter-Disciplinary Mentored Education (PRIME)	1U2CDK133422-01A1
		Improving COVID-19 Vaccine Uptake Among Racial and Ethnic Minority Groups with Rheumatic Diseases	1R01MD019235-01A1
		UAB National Coordinating Center for the George M. O'Brien Kidney National Resource Centers	U24 DK137318
		Offspring Neurodevelopment and Growth after Early-Pregnancy Antihypertensive Therapy OR Preeclampsia in Women with Chronic Hypertension (CHAP Child)	R01 HD112994
		RESTORE: Reducing future Fractures and improving Outcomes of Fragility Fracture	PCORI
3	Royalties or licenses	☒ **None**	
4	Consulting fees	☐ **None**	
		Alexion	Consulting fees
		Antisense Therapeutics	Consulting fees
		Biodelivery Sciences International	Consulting fees
		Biogen	Consulting fees
		Clinical Trail Solutions LLC	Consulting fees
		Genentech	Consulting fees
		GW Pharmaceuticals	Consulting fees
		Immunic	Consulting fees
		Klein-Buendel Incorporated	Consulting fees
		Medimmune/Viela Bio	Consulting fees
		Medday	Consulting fees
		Merck/Serono	Consulting fees
		Neurogenesis LTD	Consulting fees
		Osmotica Pharmaceuticals	Consulting fees
		Perception Neurosciences	Consulting fees

		Name all entities with whom you have this relationship or indicate none (add rows as needed)	Specifications/Comments (e.g., if payments were made to you or to your institution)
		Reckover Pharmaceuticals	Consulting fees
		Recursion/Cerexis Pharmaceuticals	Consulting fees
		Regeneron	Consulting fees
		Roche	Consulting fees
		SAB Biotherapeutics	Consulting fees
		Sanofi	Consulting fees
		TG Therapeutics	Consulting fees
		Avotres Therapeutics	Consulting fees
		Genzyme	Consulting fees
		Hoya	Consulting fees
		Linical	Consulting fees
		Immunosis Pty Ltd	Consulting fees
5	Payment or honoraria for lectures, presentations, speakers bureaus, manuscript writing or educational events	☒ **None**	
6	Payment for expert testimony	☐ **None**	
		Scott & Scott	Statistical advice on Psychosis suit – Parkinsons, Alzheimers and misc. etiologies
7	Support for attending meetings and/or travel	☐ **None**	
		Roche for Steering Committee	Travel
8	Patents planned, issued or pending	☒ **None**	
9	Participation on a Data Safety Monitoring Board or Advisory Board	☐ **None**	
		Alexion	Advisory Board
		Antisense Therapeutics	Advisory Board
		Astra Zeneca	Data and Safety Monitoring Board
		Avexis Pharmaceuticals	Data and Safety Monitoring Board
		Biodelivery Sciences International	Advisory Board
		Biogen	Advisory Board
		Biolinerx	Data and Safety Monitoring Board
		Brainstorm Cell Therapeutics	Data and Safety Monitoring Board

		Name all entities with whom you have this relationship or indicate none (add rows as needed)	Specifications/Comments (e.g., if payments were made to you or to your institution)
		Bristol Myers Squibb/Celgene	Data and Safety Monitoring Board
		Clinical Trail Solutions LLC	Advisory Board
		CSL Behring	Data and Safety Monitoring Board
		Galmed Pharmaceuticals	Data and Safety Monitoring Board
		Genentech	Advisory Board
		Green Valley Pharma	Data and Safety Monitoring Board
		GW Pharmaceuticals	Advisory Board
		Immunic	Advisory Board
		Klein-Buendel Incorporated	Advisory Board
		Mapi Pharmaceuticals LTD	Data and Safety Monitoring Board
		Medimmune/Viela Bio	Advisory Board
		Medday	Advisory Board
		Merck/Serono	Advisory Board
		Merck	Data and Safety Monitoring Board
		Merck/Pfizer	Data and Safety Monitoring Board
		Mitsubishi Tanabe Pharma Holdings	Data and Safety Monitoring Board
		Neurim	Data and Safety Monitoring Board
		Neurogenesis LTD	Advisory Board
		Novartis	Data and Safety Monitoring Board
		Ophazyme	Data and Safety Monitoring Board
		Opko Biologics	Data and Safety Monitoring Board
		Osmotica Pharmaceuticals	Advisory Board
		Perception Neurosciences	Advisory Board
		Reata Pharmaceuticals	Data and Safety Monitoring Board
		Reckover Pharmaceuticals	Advisory Board
		Recursion/Cerexis Pharmaceuticals	Advisory Board
		Regeneron	Advisory Board
		Roche	Advisory Board
		SAB Biotherapeutics	Advisory Board
		Sanofi	Advisory Board/Data and Safety Monitoring Board
		Teva Pharmaceuticals	Data and Safety Monitoring Board
		TG Therapeutics	Advisory Board
		VielaBio Inc.	Data and Safety Monitoring Board
		Applied Therapeutics	Data and Safety Monitoring Board
		AI Therapeutics	Data and Safety Monitoring Board
		AMO Pharmaceuticals	Data and Safety Monitoring Board
**10**	Leadership or fiduciary role in other board, society, committee or advocacy group, paid or unpaid	☐ **None**	
		Consortium of MS Centers	Board Member
		Birmingham Jewish Foundation	Board Member
		Birmingham Jewish Federation	Board Member
**11**	Stock or stock options	☒ **None**	

		Name all entities with whom you have this relationship or indicate none (add rows as needed)	Specifications/Comments (e.g., if payments were made to you or to your institution)
**12**	Receipt of equipment, materials, drugs, medical writing, gifts or other services	☒ **None**	
**13**	Other financial or non-financial interests	☒ **None**	

Please place an "X" next to the following statement to indicate your agreement:

☒ I certify that I have answered every question and have not altered the wording of any of the questions on this form.

ICMJE DISCLOSURE FORM

**Date:**	4/15/2024
**Your Name:**	Neil P. Oxtoby
**Manuscript Title:**	Statistical Considerations When Estimating Time-Saving Treatment Effects in Alzheimer's Clinical Trials
**Manuscript Number (if known):**	ADJ-D-24-00175

In the interest of transparency, we ask you to disclose all relationships/activities/interests listed below that are related to the content of your manuscript. "Related" means any relation with for-profit or not-for-profit third parties whose interests may be affected by the content of the manuscript. Disclosure represents a commitment to transparency and does not necessarily indicate a bias. If you are in doubt about whether to list a relationship/activity/interest, it is preferable that you do so.

The author's relationships/activities/interests should be defined broadly. For example, if your manuscript pertains to the epidemiology of hypertension, you should declare all relationships with manufacturers of antihypertensive medication, even if that medication is not mentioned in the manuscript.

In item #1 below, report all support for the work reported in this manuscript without time limit. For all other items, the time frame for disclosure is the past 36 months.

		**Name all entities with whom you have this relationship or indicate none (add rows as needed)**	**Specifications/Comments (e.g., if payments were made to you or to your institution)**
**Time frame: Since the initial planning of the work**			
1	All support for the present manuscript (e.g., funding, provision of study materials, medical writing, article processing charges, etc.) **No time limit for this item.**	☐ None	
		UKRI Medical Research Council	Research Fellowship paid to institution
			Click the tab key to add additional rows.
**Time frame: past 36 months**			
**2**	Grants or contracts from any entity (if not indicated in item #1 above).	☒ **None**	
**3**	Royalties or licenses	☒ **None**	

		Name all entities with whom you have this relationship or indicate none (add rows as needed)	Specifications/Comments (e.g., if payments were made to you or to your institution)
**4**	Consulting fees	☐ **None**	
		Therapanacea (FR)	Personal fees
		Queen Square Analytics Limited (UK)	Personal fees
**5**	Payment or honoraria for lectures, presentations, speakers bureaus, manuscript writing or educational events	☒ **None**	
**6**	Payment for expert testimony	☒ **None**	
**7**	Support for attending meetings and/or travel	☒ **None**	
**8**	Patents planned, issued or pending	☒ **None**	
**9**	Participation on a Data Safety Monitoring Board or Advisory Board	☒ **None**	
**10**	Leadership or fiduciary role in other board, society, committee or advocacy group, paid or unpaid	☐ **None**	
		DEMON Network	Unpaid

		Name all entities with whom you have this relationship or indicate none (add rows as needed)	Specifications/Comments (e.g., if payments were made to you or to your institution)
**11**	Stock or stock options	☐ **None**	
		Queen Square Analytics Limited (UK)	Options
**12**	Receipt of equipment, materials, drugs, medical writing, gifts or other services	☒ **None**	
**13**	Other financial or non-financial interests	☒ **None**	

Please place an "X" next to the following statement to indicate your agreement:

☒ I certify that I have answered every question and have not altered the wording of any of the questions on this form.

ICMJE DISCLOSURE FORM

Date: 4/5/2024

Your Name: Guogen Shan

Manuscript Title: Statistical Considerations When Estimating Time-Saving Treatment Effects in Alzheimer's Clinical Trials

Manuscript Number (if known): ADJ-D-24-00175

In the interest of transparency, we ask you to disclose all relationships/activities/interests listed below that are related to the content of your manuscript. "Related" means any relation with for-profit or not-for-profit third parties whose interests may be affected by the content of the manuscript. Disclosure represents a commitment to transparency and does not necessarily indicate a bias. If you are in doubt about whether to list a relationship/activity/interest, it is preferable that you do so.

The author's relationships/activities/interests should be defined broadly. For example, if your manuscript pertains to the epidemiology of hypertension, you should declare all relationships with manufacturers of antihypertensive medication, even if that medication is not mentioned in the manuscript.

In item #1 below, report all support for the work reported in this manuscript without time limit. For all other items, the time frame for disclosure is the past 36 months.

		**Name all entities with whom you have this relationship or indicate none (add rows as needed)**	**Specifications/Comments (e.g., if payments were made to you or to your institution)**
		Time frame: Since the initial planning of the work	
1	All support for the present manuscript (e.g., funding, provision of study materials, medical writing, article processing charges, etc.) **No time limit for this item.**	☒ None	
			Click the tab key to add additional rows.
		Time frame: past 36 months	
**2**	Grants or contracts from any entity (if not indicated in item #1 above).	☒ **None**	
**3**	Royalties or licenses	☒ **None**	

		Name all entities with whom you have this relationship or indicate none (add rows as needed)	Specifications/Comments (e.g., if payments were made to you or to your institution)
**4**	Consulting fees	☒ **None**	
**5**	Payment or honoraria for lectures, presentations, speakers bureaus, manuscript writing or educational events	☒ **None**	
**6**	Payment for expert testimony	☒ **None**	
**7**	Support for attending meetings and/or travel	☒ **None**	
**8**	Patents planned, issued or pending	☒ **None**	
**9**	Participation on a Data Safety Monitoring Board or Advisory Board	☒ **None**	
**10**	Leadership or fiduciary role in other board, society, committee or advocacy group, paid or unpaid	☒ **None**	

		Name all entities with whom you have this relationship or indicate none (add rows as needed)	Specifications/Comments (e.g., if payments were made to you or to your institution)
**11**	Stock or stock options	☒ **None**	
**12**	Receipt of equipment, materials, drugs, medical writing, gifts or other services	☒ **None**	
**13**	Other financial or non-financial interests	☒ **None**	

Please place an "X" next to the following statement to indicate your agreement:

☒ I certify that I have answered every question and have not altered the wording of any of the questions on this form.

ICMJE DISCLOSURE FORM

**Date:**	4/12/2024
**Your Name:**	Whedy Wang
**Manuscript Title:**	Statistical Considerations When Estimating Time-Saving Treatment Effects in Alzheimer's Clinical Trials
**Manuscript Number (if known):**	ADJ-D-24-00175

In the interest of transparency, we ask you to disclose all relationships/activities/interests listed below that are related to the content of your manuscript. "Related" means any relation with for-profit or not-for-profit third parties whose interests may be affected by the content of the manuscript. Disclosure represents a commitment to transparency and does not necessarily indicate a bias. If you are in doubt about whether to list a relationship/activity/interest, it is preferable that you do so.

The author's relationships/activities/interests should be defined broadly. For example, if your manuscript pertains to the epidemiology of hypertension, you should declare all relationships with manufacturers of antihypertensive medication, even if that medication is not mentioned in the manuscript.

In item #1 below, report all support for the work reported in this manuscript without time limit. For all other items, the time frame for disclosure is the past 36 months.

		**Name all entities with whom you have this relationship or indicate none (add rows as needed)**	**Specifications/Comments (e.g., if payments were made to you or to your institution)**
**Time frame: Since the initial planning of the work**			
1	All support for the present manuscript (e.g., funding, provision of study materials, medical writing, article processing charges, etc.) **No time limit for this item.**	☒ None	
			Click the tab key to add additional rows.
**Time frame: past 36 months**			
**2**	Grants or contracts from any entity (if not indicated in item #1 above).	☒ **None**	
**3**	Royalties or licenses	☒ **None**	

		Name all entities with whom you have this relationship or indicate none (add rows as needed)	Specifications/Comments (e.g., if payments were made to you or to your institution)
**4**	Consulting fees	☒ **None**	
**5**	Payment or honoraria for lectures, presentations, speakers bureaus, manuscript writing or educational events	☒ **None**	
**6**	Payment for expert testimony	☒ **None**	
**7**	Support for attending meetings and/or travel	☒ **None**	
**8**	Patents planned, issued or pending	☒ **None**	
**9**	Participation on a Data Safety Monitoring Board or Advisory Board	☒ **None**	
**10**	Leadership or fiduciary role in other board, society, committee or advocacy group, paid or unpaid	☒ **None**	

		Name all entities with whom you have this relationship or indicate none (add rows as needed)	Specifications/Comments (e.g., if payments were made to you or to your institution)
**11**	Stock or stock options	☒ **None**	
**12**	Receipt of equipment, materials, drugs, medical writing, gifts or other services	☒ **None**	
**13**	Other financial or non-financial interests	☒ **None**	

Please place an "X" next to the following statement to indicate your agreement:

☒ I certify that I have answered every question and have not altered the wording of any of the questions on this form.

ICMJE DISCLOSURE FORM

**Date:**	4/15/2024
**Your Name:**	Brian Mangal
**Manuscript Title:**	Statistical Considerations When Estimating Time-Saving Treatment Effects in Alzheimer's Clinical Trials
**Manuscript Number (if known):**	ADJ-D-24-00175

In the interest of transparency, we ask you to disclose all relationships/activities/interests listed below that are related to the content of your manuscript. "Related" means any relation with for-profit or not-for-profit third parties whose interests may be affected by the content of the manuscript. Disclosure represents a commitment to transparency and does not necessarily indicate a bias. If you are in doubt about whether to list a relationship/activity/interest, it is preferable that you do so.

The author's relationships/activities/interests should be defined broadly. For example, if your manuscript pertains to the epidemiology of hypertension, you should declare all relationships with manufacturers of antihypertensive medication, even if that medication is not mentioned in the manuscript.

In item #1 below, report all support for the work reported in this manuscript without time limit. For all other items, the time frame for disclosure is the past 36 months.

		**Name all entities with whom you have this relationship or indicate none (add rows as needed)**	**Specifications/Comments (e.g., if payments were made to you or to your institution)**
**Time frame: Since the initial planning of the work**			
1	All support for the present manuscript (e.g., funding, provision of study materials, medical writing, article processing charges, etc.) **No time limit for this item.**	☒ None	
			Click the tab key to add additional rows.
**Time frame: past 36 months**			
**2**	Grants or contracts from any entity (if not indicated in item #1 above).	☒ **None**	
**3**	Royalties or licenses	☒ **None**	

		Name all entities with whom you have this relationship or indicate none (add rows as needed)	Specifications/Comments (e.g., if payments were made to you or to your institution)
**4**	Consulting fees	☒ **None**	
**5**	Payment or honoraria for lectures, presentations, speakers bureaus, manuscript writing or educational events	☒ **None**	
**6**	Payment for expert testimony	☒ **None**	
**7**	Support for attending meetings and/or travel	☒ **None**	
**8**	Patents planned, issued or pending	☒ **None**	
**9**	Participation on a Data Safety Monitoring Board or Advisory Board	☒ **None**	
**10**	Leadership or fiduciary role in other board, society, committee or advocacy group, paid or unpaid	☒ **None**	

		Name all entities with whom you have this relationship or indicate none (add rows as needed)	Specifications/Comments (e.g., if payments were made to you or to your institution)
**11**	Stock or stock options	☒ **None**	
**12**	Receipt of equipment, materials, drugs, medical writing, gifts or other services	☒ **None**	
**13**	Other financial or non-financial interests	☒ **None**	

Please place an "X" next to the following statement to indicate your agreement:

☒ I certify that I have answered every question and have not altered the wording of any of the questions on this form.

ICMJE DISCLOSURE FORM

Date: 4/11/2024

Your Name: Yijie Liao

Manuscript Title: Statistical Considerations When Estimating Time-Saving Treatment Effects in Alzheimer's Clinical Trials

Manuscript Number (if known): ADJ-D-24-00175

In the interest of transparency, we ask you to disclose all relationships/activities/interests listed below that are related to the content of your manuscript. "Related" means any relation with for-profit or not-for-profit third parties whose interests may be affected by the content of the manuscript. Disclosure represents a commitment to transparency and does not necessarily indicate a bias. If you are in doubt about whether to list a relationship/activity/interest, it is preferable that you do so.

The author's relationships/activities/interests should be defined broadly. For example, if your manuscript pertains to the epidemiology of hypertension, you should declare all relationships with manufacturers of antihypertensive medication, even if that medication is not mentioned in the manuscript.

In item #1 below, report all support for the work reported in this manuscript without time limit. For all other items, the time frame for disclosure is the past 36 months.

		Name all entities with whom you have this relationship or indicate none (add rows as needed)	Specifications/Comments (e.g., if payments were made to you or to your institution)
Time frame: Since the initial planning of the work			
1	All support for the present manuscript (e.g., funding, provision of study materials, medical writing, article processing charges, etc.) No time limit for this item.	☒ None	
			Click the tab key to add additional rows.
Time frame: past 36 months			
**2**	Grants or contracts from any entity (if not indicated in item #1 above).	☒ **None**	
**3**	Royalties or licenses	☒ **None**	

		Name all entities with whom you have this relationship or indicate none (add rows as needed)	Specifications/Comments (e.g., if payments were made to you or to your institution)
**4**	Consulting fees	☒ **None**	
**5**	Payment or honoraria for lectures, presentations, speakers bureaus, manuscript writing or educational events	☒ **None**	
**6**	Payment for expert testimony	☒ **None**	
**7**	Support for attending meetings and/or travel	☒ **None**	
**8**	Patents planned, issued or pending	☒ **None**	
**9**	Participation on a Data Safety Monitoring Board or Advisory Board	☒ **None**	
**10**	Leadership or fiduciary role in other board, society, committee or advocacy group, paid or unpaid	☒ **None**	

		Name all entities with whom you have this relationship or indicate none (add rows as needed)	Specifications/Comments (e.g., if payments were made to you or to your institution)
**11**	Stock or stock options	☒ **None**	
**12**	Receipt of equipment, materials, drugs, medical writing, gifts or other services	☒ **None**	
**13**	Other financial or non-financial interests	☒ **None**	

Please place an "X" next to the following statement to indicate your agreement:

☒ I certify that I have answered every question and have not altered the wording of any of the questions on this form.

ICMJE DISCLOSURE FORM

Date: 4/7/2024

Your Name: Jorge J Llibre Guerra

Manuscript Title: Statistical Considerations When Estimating Time-Saving Treatment Effects in Alzheimer's Clinical Trials

Manuscript Number (if known): ADJ-D-24-00175

In the interest of transparency, we ask you to disclose all relationships/activities/interests listed below that are related to the content of your manuscript. "Related" means any relation with for-profit or not-for-profit third parties whose interests may be affected by the content of the manuscript. Disclosure represents a commitment to transparency and does not necessarily indicate a bias. If you are in doubt about whether to list a relationship/activity/interest, it is preferable that you do so.

The author's relationships/activities/interests should be defined broadly. For example, if your manuscript pertains to the epidemiology of hypertension, you should declare all relationships with manufacturers of antihypertensive medication, even if that medication is not mentioned in the manuscript.

In item #1 below, report all support for the work reported in this manuscript without time limit. For all other items, the time frame for disclosure is the past 36 months.

		**Name all entities with whom you have this relationship or indicate none (add rows as needed)**	**Specifications/Comments (e.g., if payments were made to you or to your institution)**
		Time frame: Since the initial planning of the work	
1	All support for the present manuscript (e.g., funding, provision of study materials, medical writing, article processing charges, etc.) **No time limit for this item.**	☒ None	
			Click the tab key to add additional rows.
		Time frame: past 36 months	
2	Grants or contracts from any entity (if not indicated in item #1 above).	☐ **None**	
		K01AG073526	
		SG-20-690363	
		AARFD-21-851415	
3	Royalties or licenses	☒ **None**	

		Name all entities with whom you have this relationship or indicate none (add rows as needed)	Specifications/Comments (e.g., if payments were made to you or to your institution)
**4**	Consulting fees	☒ **None**	
**5**	Payment or honoraria for lectures, presentations, speakers bureaus, manuscript writing or educational events	☒ **None**	
**6**	Payment for expert testimony	☒ **None**	
**7**	Support for attending meetings and/or travel	☒ **None**	
**8**	Patents planned, issued or pending	☒ **None**	
**9**	Participation on a Data Safety Monitoring Board or Advisory Board	☒ **None**	
**10**	Leadership or fiduciary role in other board, society, committee or advocacy group, paid or unpaid	☒ **None**	

		Name all entities with whom you have this relationship or indicate none (add rows as needed)	Specifications/Comments (e.g., if payments were made to you or to your institution)
**11**	Stock or stock options	☒ **None**	
**12**	Receipt of equipment, materials, drugs, medical writing, gifts or other services	☒ **None**	
**13**	Other financial or non-financial interests	☒ **None**	

Please place an "X" next to the following statement to indicate your agreement:

☒ I certify that I have answered every question and have not altered the wording of any of the questions on this form.

ICMJE DISCLOSURE FORM

Date: 4/8/2024

Your Name: Yan Li

Manuscript Title: Statistical Considerations When Estimating Time-Saving Treatment Effects in Alzheimer's Clinical Trials

Manuscript Number (if known): ADJ-D-24-00175

In the interest of transparency, we ask you to disclose all relationships/activities/interests listed below that are related to the content of your manuscript. "Related" means any relation with for-profit or not-for-profit third parties whose interests may be affected by the content of the manuscript. Disclosure represents a commitment to transparency and does not necessarily indicate a bias. If you are in doubt about whether to list a relationship/activity/interest, it is preferable that you do so.

The author's relationships/activities/interests should be defined broadly. For example, if your manuscript pertains to the epidemiology of hypertension, you should declare all relationships with manufacturers of antihypertensive medication, even if that medication is not mentioned in the manuscript.

In item #1 below, report all support for the work reported in this manuscript without time limit. For all other items, the time frame for disclosure is the past 36 months.

		Name all entities with whom you have this relationship or indicate none (add rows as needed)	Specifications/Comments (e.g., if payments were made to you or to your institution)
Time frame: Since the initial planning of the work			
1	All support for the present manuscript (e.g., funding, provision of study materials, medical writing, article processing charges, etc.) **No time limit for this item.**	☒ None	
			Click the tab key to add additional rows.
Time frame: past 36 months			
**2**	Grants or contracts from any entity (if not indicated in item #1 above).	☒ **None**	
**3**	Royalties or licenses	☒ **None**	

		Name all entities with whom you have this relationship or indicate none (add rows as needed)	Specifications/Comments (e.g., if payments were made to you or to your institution)
**4**	Consulting fees	☒ **None**	
**5**	Payment or honoraria for lectures, presentations, speakers bureaus, manuscript writing or educational events	☒ **None**	
**6**	Payment for expert testimony	☒ **None**	
**7**	Support for attending meetings and/or travel	☒ **None**	
**8**	Patents planned, issued or pending	☒ **None**	
**9**	Participation on a Data Safety Monitoring Board or Advisory Board	☒ **None**	
**10**	Leadership or fiduciary role in other board, society, committee or advocacy group, paid or unpaid	☒ **None**	

		Name all entities with whom you have this relationship or indicate none (add rows as needed)	Specifications/Comments (e.g., if payments were made to you or to your institution)
**11**	Stock or stock options	☒ **None**	
**12**	Receipt of equipment, materials, drugs, medical writing, gifts or other services	☒ **None**	
**13**	Other financial or non-financial interests	☒ **None**	

Please place an "X" next to the following statement to indicate your agreement:

☒ I certify that I have answered every question and have not altered the wording of any of the questions on this form.

ICMJE DISCLOSURE FORM

Date: 4/8/2024

Your Name: Chengjie Xiong

Manuscript Title: Statistical Considerations When Estimating Time-Saving Treatment Effects in Alzheimer's Clinical Trials

Manuscript Number (if known): ADJ-D-24-00175

In the interest of transparency, we ask you to disclose all relationships/activities/interests listed below that are related to the content of your manuscript. "Related" means any relation with for-profit or not-for-profit third parties whose interests may be affected by the content of the manuscript. Disclosure represents a commitment to transparency and does not necessarily indicate a bias. If you are in doubt about whether to list a relationship/activity/interest, it is preferable that you do so.

The author's relationships/activities/interests should be defined broadly. For example, if your manuscript pertains to the epidemiology of hypertension, you should declare all relationships with manufacturers of antihypertensive medication, even if that medication is not mentioned in the manuscript.

In item #1 below, report all support for the work reported in this manuscript without time limit. For all other items, the time frame for disclosure is the past 36 months.

		Name all entities with whom you have this relationship or indicate none (add rows as needed)	Specifications/Comments (e.g., if payments were made to you or to your institution)
Time frame: Since the initial planning of the work			
1	All support for the present manuscript (e.g., funding, provision of study materials, medical writing, article processing charges, etc.) **No time limit for this item.**	☐ None	
		NIH Grant AG067505	Dr. Chengjie Xiong
			Click the tab key to add additional rows.
Time frame: past 36 months			
2	Grants or contracts from any entity (if not indicated in item #1 above).	☒ **None**	
3	Royalties or licenses	☒ **None**	

		Name all entities with whom you have this relationship or indicate none (add rows as needed)	Specifications/Comments (e.g., if payments were made to you or to your institution)
**4**	Consulting fees	☐ **None**	
		Diadem	Payment to Chengjie Xiong
**5**	Payment or honoraria for lectures, presentations, speakers bureaus, manuscript writing or educational events	☒ **None**	
**6**	Payment for expert testimony	☒ **None**	
**7**	Support for attending meetings and/or travel	☒ **None**	
**8**	Patents planned, issued or pending	☒ **None**	
**9**	Participation on a Data Safety Monitoring Board or Advisory Board	☐ **None**	
		FDA Advisory Committee on Imaging Medical Products	
**10**	Leadership or fiduciary role in other board, society, committee or advocacy group, paid or unpaid	☒ **None**	

		Name all entities with whom you have this relationship or indicate none (add rows as needed)	Specifications/Comments (e.g., if payments were made to you or to your institution)
**11**	Stock or stock options	☒ **None**	
**12**	Receipt of equipment, materials, drugs, medical writing, gifts or other services	☒ **None**	
**13**	Other financial or non-financial interests	☒ **None**	
		C2N Diagnostics	Work citing NIH grant #AG067505 requires an institutional disclosure. Washington University School of Medicine in St. Louis has a financial interest in C2N Diagnostics and may financially benefit if the company is successful in marketing its product(s) that are related to this research. The current study is not directly concerned by this statement as it does not utilize data from this project.

Please place an "X" next to the following statement to indicate your agreement:

☒ I certify that I have answered every question and have not altered the wording of any of the questions on this form.

ICMJE DISCLOSURE FORM

**Date:**	4/11/2024
**Your Name:**	Eric McDade
**Manuscript Title:**	Statistical Considerations When Estimating Time-Saving Treatment Effects in Alzheimer's Clinical Trials
**Manuscript Number (if known):**	ADJ-D-24-00175

In the interest of transparency, we ask you to disclose all relationships/activities/interests listed below that are related to the content of your manuscript. "Related" means any relation with for-profit or not-for-profit third parties whose interests may be affected by the content of the manuscript. Disclosure represents a commitment to transparency and does not necessarily indicate a bias. If you are in doubt about whether to list a relationship/activity/interest, it is preferable that you do so.

The author's relationships/activities/interests should be defined broadly. For example, if your manuscript pertains to the epidemiology of hypertension, you should declare all relationships with manufacturers of antihypertensive medication, even if that medication is not mentioned in the manuscript.

In item #1 below, report all support for the work reported in this manuscript without time limit. For all other items, the time frame for disclosure is the past 36 months.

		**Name all entities with whom you have this relationship or indicate none (add rows as needed)**	**Specifications/Comments (e.g., if payments were made to you or to your institution)**
		**Time frame: Since the initial planning of the work**	
1	All support for the present manuscript (e.g., funding, provision of study materials, medical writing, article processing charges, etc.) **No time limit for this item.**	☒ None	
			Click the tab key to add additional rows.
		**Time frame: past 36 months**	
**2**	Grants or contracts from any entity (if not indicated in item #1 above).	☐ **None**	
		NIA	Payment to Institution
		Eli Lilly	Payment to Institution
		Roche	Payment to Institution
		Eisa	Payment to Institution
		GHR	Payment to Institution
**3**	Royalties or licenses	☒ **None**	

		Name all entities with whom you have this relationship or indicate none (add rows as needed)	Specifications/Comments (e.g., if payments were made to you or to your institution)
**4**	Consulting fees	☐ **None**	
		Astra Zeneca	Paid to me
		Roche	Paid to me
		Sanofi	Paid to me
		Merck	Paid to me
**5**	Payment or honoraria for lectures, presentations, speakers bureaus, manuscript writing or educational events	☐ **None**	
		Alzheimer Association	Manuscript preparation
		Projects in Knowledge (Kaplan)- CME	Paid to me
		Neurology Live- CME	Paid to me
**6**	Payment for expert testimony	☒ **None**	
**7**	Support for attending meetings and/or travel	☐ **None**	
		Alzheimer Association	
		Fondation Alzheimer	
**8**	Patents planned, issued or pending	☒ **None**	
**9**	Participation on a Data Safety Monitoring Board or Advisory Board	☐ **None**	
		Alector	Paid to me
**10**	Leadership or fiduciary role in other board, society, committee or advocacy group, paid or unpaid	☐ **None**	
		Alzamend	Paid to me

		Name all entities with whom you have this relationship or indicate none (add rows as needed)	Specifications/Comments (e.g., if payments were made to you or to your institution)
**11**	Stock or stock options	☒ **None**	
**12**	Receipt of equipment, materials, drugs, medical writing, gifts or other services	☒ **None**	
**13**	Other financial or non-financial interests	☒ **None**	

Please place an "X" next to the following statement to indicate your agreement:

☒ I certify that I have answered every question and have not altered the wording of any of the questions on this form.

ICMJE DISCLOSURE FORM

**Date:**	4/11/2024
**Your Name:**	Paul Delmar
**Manuscript Title:**	Statistical Considerations When Estimating Time-Saving Treatment Effects in Alzheimer's Clinical Trials
**Manuscript Number (if known):**	ADJ-D-24-00175

In the interest of transparency, we ask you to disclose all relationships/activities/interests listed below that are related to the content of your manuscript. "Related" means any relation with for-profit or not-for-profit third parties whose interests may be affected by the content of the manuscript. Disclosure represents a commitment to transparency and does not necessarily indicate a bias. If you are in doubt about whether to list a relationship/activity/interest, it is preferable that you do so.

The author's relationships/activities/interests should be defined broadly. For example, if your manuscript pertains to the epidemiology of hypertension, you should declare all relationships with manufacturers of antihypertensive medication, even if that medication is not mentioned in the manuscript.

In item #1 below, report all support for the work reported in this manuscript without time limit. For all other items, the time frame for disclosure is the past 36 months.

		**Name all entities with whom you have this relationship or indicate none (add rows as needed)**	**Specifications/Comments (e.g., if payments were made to you or to your institution)**
		Time frame: Since the initial planning of the work	
1	All support for the present manuscript (e.g., funding, provision of study materials, medical writing, article processing charges, etc.) **No time limit for this item.**	☒ None	
			Click the tab key to add additional rows.
		Time frame: past 36 months	
2	Grants or contracts from any entity (if not indicated in item #1 above).	☒ **None**	
3	Royalties or licenses	☒ **None**	

		Name all entities with whom you have this relationship or indicate none (add rows as needed)	Specifications/Comments (e.g., if payments were made to you or to your institution)
**4**	Consulting fees	☒ **None**	
**5**	Payment or honoraria for lectures, presentations, speakers bureaus, manuscript writing or educational events	☒ **None**	
**6**	Payment for expert testimony	☒ **None**	
**7**	Support for attending meetings and/or travel	☒ **None**	
**8**	Patents planned, issued or pending	☒ **None**	
**9**	Participation on a Data Safety Monitoring Board or Advisory Board	☒ **None**	
**10**	Leadership or fiduciary role in other board, society, committee or advocacy group, paid or unpaid	☒ **None**	

		Name all entities with whom you have this relationship or indicate none (add rows as needed)	Specifications/Comments (e.g., if payments were made to you or to your institution)
**11**	Stock or stock options	☐ **None**	
		F. Hoffmann - La Roche Ltd	
**12**	Receipt of equipment, materials, drugs, medical writing, gifts or other services	☒ **None**	
**13**	Other financial or non-financial interests	☐ **None**	
		Full time employee of F. Hoffmann - La Roche Ltd	

Please place an "X" next to the following statement to indicate your agreement:

☒ I certify that I have answered every question and have not altered the wording of any of the questions on this form.

ICMJE DISCLOSURE FORM

Date: 4/8/2024

Your Name: Randall Bateman

Manuscript Title: Examining Amyloid Reduction as A Surrogate Endpoint through Latent Class Analysis Using Clinical Trial Data for Dominantly Inherited Alzheimer's Disease

Manuscript Number (if known): ADJ-D-23-1203

In the interest of transparency, we ask you to disclose all relationships/activities/interests listed below that are related to the content of your manuscript. "Related" means any relation with for-profit or not-for-profit third parties whose interests may be affected by the content of the manuscript. Disclosure represents a commitment to transparency and does not necessarily indicate a bias. If you are in doubt about whether to list a relationship/activity/interest, it is preferable that you do so.

The author's relationships/activities/interests should be defined broadly. For example, if your manuscript pertains to the epidemiology of hypertension, you should declare all relationships with manufacturers of antihypertensive medication, even if that medication is not mentioned in the manuscript.

In item #1 below, report all support for the work reported in this manuscript without time limit. For all other items, the time frame for disclosure is the past 36 months.

		Name all entities with whom you have this relationship or indicate none (add rows as needed)	Specifications/Comments (e.g., if payments were made to you or to your institution)
		Time frame: Since the initial planning of the work	
1	All support for the present manuscript (e.g., funding, provision of study materials, medical writing, article processing charges, etc.) **No time limit for this item.**	☐ None	
		National Institute on Aging U01AG042791, FNIH/AMP U01AG42791-S1	PI: Randall Bateman Dominantly Inherited Alzheimer Network (DIAN) Trial–An Opportunity to Prevent Dementia - Research Grant
		National Institute on Aging R01AG046179	PI: Randall Bateman Dominantly Inherited Alzheimer's Network Trials Unit-Adaptive Prevention Trial - Research Grant
		National Institute on Aging R01AG53627/R56AG53627	PI: Randall Bateman DIAN-TU Next Generation Prevention Trial - Research Grant
		GHR Foundation	PI: Randall Bateman Dominantly Inherited Alzheimer Network (DIAN) Trials Unit Sustainable Funding – Research Grant
		Alzheimer's Association<br><br>DIAN-TTU-12-243040<br>DIAN TU NG-16-434362	PI: Randall Bateman<br>Dominantly Inherited Alzheimer Network – Therapeutic Treatment Unit (TTU) Grant<br>DIAN-TU Next Generation Grant Trial – Research Grant
		DIAN-TU Pharma Consortium	Active: Eli Lilly and Company/Avid Radiopharmaceuticals, Hoffman-La Roche/Genentech, Biogen, Eisai, Janssen. Previous: Abbvie, Amgen, AstraZeneca, Forum, Mithridion, Novartis, Pfizer, United Neuroscience, Sanofi).
		Avid Pharmaceuticals	Receipt of tracer.
		Eli Lilly and Company	Receipt of drugs and services. Tau SILK Consortium Member.

		Name all entities with whom you have this relationship or indicate none (add rows as needed)	Specifications/Comments (e.g., if payments were made to you or to your institution)
		Hoffman-La Roche	Receipt of drugs and services. NfL Consortium Member.
		Anonymous Foundation	PI: Randall Bateman Dominantly Inherited Alzheimer Network – Therapeutic Treatment Unit (TTU) Research Grant
		CogState	In-kind support
		Signant	In-kind support
Time frame: past 36 months			
2	Grants or contracts from any entity (if not indicated in item #1 above).	☐ **None**	
		National Institute on Aging R01AG068319	PI: Randall Bateman<br>DIAN-TU Next Generation Tau Trial - grant
		Alzheimer's Association<br>DIAN-TU-OLE-21-725093<br>DIAN-TU-Tau-21-822987,	PI: Randall Bateman<br>DIAN-TU Open Label Extension – grant<br>DIAN-TU Tau Next Generation - grant
		Biogen	Tau SILK Consortium member<br>NfL Consortium member
		AbbVie	Tau SILK Consortium member<br>NfL Consortium member
		Bristol Meyer Squibbs	NfL Consortium member
		Novartis	Tau SILK Consortium member
		National Institute on Aging UFAG032438	PI: Randall Bateman, DIAN - grant
		National Institute on Aging RF1AG061900, R56AG061900	PI: Randall Bateman, Blood AB - grant
		National Institute on Aging R21AG067559	PI: Randall Bateman, NfL - grant
		NINDS/NIA R01NS095773	PI: Randall Bateman, CNS Tau - grant
		Centene Corporation	Investigator Initiated Research - grant
		Rainwater Foundation	Investigator Initiated Research - grants
		Assn for Frontotemporal Degeneration FTD Biomarkers Initiative	Investigator Initiated Research - grant
		Biogen	Investigator Initiated Research – grant
		BrightFocus Foundation	Investigator Initiated Research – grant
		Cure Alzheimer's Fund	Investigator Initiated Research – grant
		Coins for Alzheimer's Research Trust Fund	Investigator Initiated Research – grant
		Eisai	Investigator Initiated Research – grants
		The Foundation for Barnes-Jewish Hospital	Investigator Initiated Research – grant
		TargetALS	Investigator Initiated Research – grant
		Good Ventures Foundation	Investigator Initiated Research – grant

		Name all entities with whom you have this relationship or indicate none (add rows as needed)	Specifications/Comments (e.g., if payments were made to you or to your institution)
3	Royalties or licenses	☐ None	
		C2N Diagnostics	Equity ownership interest in C2N Diagnostics and receive royalty income based on technology (stable isotope labeling kinetics and blood plasma assay) licensed by Washington University to C2N Diagnostics
4	Consulting fees	☒ None	
5	Payment or honoraria for lectures, presentations, speakers bureaus, manuscript writing or educational events	☐ None	
		Korean Dementia Association	International Conference Lecture Honoraria
		American Neurological Association	Speaker - Fall Conference honoraria
		Weill Cornell Medical College	Speaker honoraria
		Fondazione Prada	Speaker Honoraria
		Harvard University	Speaker Honoraria
6	Payment for expert testimony	☒ None	
7	Support for attending meetings and/or travel	☐ None	
		Alzheimer's Association Roundtable	Reimbursed for travel expenses
		Duke Margolis Alzheimer's Roundtable	Reimbursed for travel expenses
		BrightFocus Foundation	Reimbursed for travel expenses
		Tau Consortium Investigator's Meeting	Reimbursed for travel expenses
		NAPA Advisory Council on Alzheimer's Research	Reimbursed for lodging & ground transportation
		CTAD Annual Meeting – Lifetime Achievement Award	Reimbursed for travel expenses for 3 days
		FBRI Meeting	Reimbursed for travel expenses
		Beeson Annual Meeting	Hotel expenses paid
		Adler Symposium	Hotel & transfers paid
8	Patents planned, issued or pending	☐ None	
		Washington University w/ RJB as coinventor - Methods for Measuring the Metabolism of CNS Derived Biomolecules In Vivo	US nonprovisional patent application 12/267,974

		Name all entities with whom you have this relationship or indicate none (add rows as needed)	Specifications/Comments (e.g., if payments were made to you or to your institution)
		Washington University w/ RJB as coinventor - Methods for Measuring the Metabolism of neurally Derived Biomolecules in vivo	US nonprovisional patent application 13/005,233
		Washington University w/ RJB as coinventor - Plasma based methods for detecting CNS Amyloid Disposition	US nonprovisional patent application 62/492,718
		Washington University w/ RJB as coinventor - Plasma based methods for determining A-Beta Amyloidosis	US nonprovisional patent application 16/610,428
		Washington University w/RJB as coinventor – Methods of Treating Based on site-specific tau phosphorylation	US nonprovisional patent application 17/015,985
		Washington University w/RJB as coinventor – Tau Kinetic Measurements	US nonprovisional patent application 15/515,909
**9**	Participation on a Data Safety Monitoring Board or Advisory Board	☐ **None**	
		Hoffman La-Roche/Genentech	Unpaid - Gantenerumab Advisory Board
		Biogen – Combination therapy for Alzheimer's disease	Unpaid Scientific Advisory Board
		UK Dementia Research Institute at University College London	Unpaid Scientific Advisory Board
		Stanford University, Next Generation Translational Proteomics for Alzheimer's and Related Dementias	Unpaid Scientific Advisory Board
**10**	Leadership or fiduciary role in other board, society, committee or advocacy group, paid or unpaid	☐ **None**	
		C2N Diagnostics	Receives income from C2N Diagnostics for serving on the scientific advisory board
**11**	Stock or stock options	☒ **None**	
**12**	Receipt of equipment, materials, drugs, medical writing, gifts or other services	☐ **None**	
		Eisai	Receipt of drugs and services, DIAN-TU Next Generation Trial
		Janssen	Receipt of drugs and services, DIAN-TU Next Generation Trial
		Hoffman La Roche	Receipt of drugs and services, DIAN-TU Open Label Extension - Gantenerumab
**13**	Other financial or non-financial interests	☒ **None**	

	Name all entities with whom you have this relationship or indicate none (add rows as needed)	Specifications/Comments (e.g., if payments were made to you or to your institution)

Please place an "X" next to the following statement to indicate your agreement:

☒ I certify that I have answered every question and have not altered the wording of any of the questions on this form.

ICMJE DISCLOSURE FORM

Date: 4/12/2024

Your Name: Lon S. Schneider

Manuscript Title: Statistical Considerations When Estimating Time-Saving Treatment Effects in Alzheimer's Clinical Trials

Manuscript Number (if known): ADJ-D-24-00175

In the interest of transparency, we ask you to disclose all relationships/activities/interests listed below that are related to the content of your manuscript. "Related" means any relation with for-profit or not-for-profit third parties whose interests may be affected by the content of the manuscript. Disclosure represents a commitment to transparency and does not necessarily indicate a bias. If you are in doubt about whether to list a relationship/activity/interest, it is preferable that you do so.

The author's relationships/activities/interests should be defined broadly. For example, if your manuscript pertains to the epidemiology of hypertension, you should declare all relationships with manufacturers of antihypertensive medication, even if that medication is not mentioned in the manuscript.

In item #1 below, report all support for the work reported in this manuscript without time limit. For all other items, the time frame for disclosure is the past 36 months.

		**Name all entities with whom you have this relationship or indicate none (add rows as needed)**	**Specifications/Comments (e.g., if payments were made to you or to your institution)**
		**Time frame: Since the initial planning of the work**	
1	All support for the present manuscript (e.g., funding, provision of study materials, medical writing, article processing charges, etc.) **No time limit for this item.**	☐ **None**	
		Della Martin Foundation	
		NIH P30 AG066530	
		NIH R01 AG051346	
		**Time frame: past 36 months**	
2	Grants or contracts from any entity (if not indicated in item #1 above).	☐ **None**	
		NIH R01 AG062687	NIH R01 AG051346
		NIH R01 AG055444	NIH P01 AG02350
		NIH R01 AG053267	NIH R01 AG074983
		NIH R01 AG063826	Biohaven
		Biogen	Eisai
		Eli Lilly	
3	Royalties or licenses	☒ **None**	

		Name all entities with whom you have this relationship or indicate none (add rows as needed)	Specifications/Comments (e.g., if payments were made to you or to your institution)
4	Consulting fees	☐ None	
		AC Immune	Cortexyme
		Alpha-cognition	BioVie
		Athira	Eli Lilly/Avid
		Corium	Lundbeck
		Merck	Novo-Nordisk
		Neurim Ltd	Otsuka
		Roche/Genentech	Cognition
		Lighthouse	GW Research
		ImmunoBrain, Lid	Bristol Myers Squibb
		Muna Ltd	Longeron
5	Payment or honoraria for lectures, presentations, speakers bureaus, manuscript writing or educational events	☒ None	
6	Payment for expert testimony	☒ None	
7	Support for attending meetings and/or travel	☐ None	
		Della Martin Foundation	
		ATRI/ACTC	
		ADRS	
8	Patents planned, issued or pending	☒ None	
9	Participation on a Data Safety Monitoring Board or Advisory Board	☒ None	
		Merck	BMS
		Genentech	
		UCB	
10	Leadership or fiduciary role in other board,	☒ None	

		Name all entities with whom you have this relationship or indicate none (add rows as needed)	Specifications/Comments (e.g., if payments were made to you or to your institution)
	society, committee or advocacy group, paid or unpaid		
**11**	Stock or stock options	☒ **None**	
**12**	Receipt of equipment, materials, drugs, medical writing, gifts or other services	☒ **None**	
**13**	Other financial or non-financial interests	☒ **None**	

Please place an "X" next to the following statement to indicate your agreement:

☒ I certify that I have answered every question and have not altered the wording of any of the questions on this form.
